# Artificial intelligence based prediction and multi-objective RSM optimization of tectona grandis biodiesel with Elaeocarpus Ganitrus

**DOI:** 10.1038/s41598-025-87640-1

**Published:** 2025-01-30

**Authors:** V Vinoth Kannan, Bhavesh Kanabar, J Gowrishankar, Ali Khatibi., Sarfaraz Kamangar, Amir Ibrahim Ali Arabi, Pushparaj Thomai, Jasmina Lozanović

**Affiliations:** 1 Indra Ganesan College of Engineering, Manikandam, Tiruchirappalli, Tamil Nadu India; 2 Department of Mechanical Engineering, Faculty of Engineering & Technology, Marwadi University Research Center, Marwadi University, Rajkot, 360003 Gujarat India; 3 Department of Computer Science Engineering, School of Engineering and Technology, JAIN (Deemed to be University), Bangalore, Karnataka India; 4 Management and Science University, Shah Alam, Selangor Malaysia; 5 Mechanical Engineering Department, College of Engineering, King Khalid University, Abha, 61421 Saudi Arabia; 6 Department of Mechanical Engineering, Kings College of Engineering, Punalkulam, Pudukkottai, Tamilnadu India; 7 Department of Engineering, FH Campus Wien - University of Applied Sciences, Favoritenstraße 226, Vienna, 1100 Austria

**Keywords:** Machine learning, Optimization, Prediction, ANN, KNN, RSM, Mechanical engineering, Energy science and technology

## Abstract

Meta-heuristic optimization algorithms are widely applied across various fields due to their intelligent behavior and fast convergence, but their use in optimizing engine behavior remains limited. This study addresses this gap by integrating the Design of Experiments-based Response Surface Methodology (RSM) with meta-heuristic optimization techniques to enhance engine performance and emissions characteristics using Tectona Grandi’s biodiesel with Elaeocarpus Ganitrus as an additive. Advanced Machine Learning (ML) models, including Artificial Neural Networks (ANN), K-Nearest Neighbors (KNN), Extreme Gradient Boosting (XGB), and Random Trees (RT), were employed for predictive analysis, with ANN outperforming RSM in accuracy. The study identified the Teak biodiesel blend (TB20) with a 5 ml Elaeocarpus Ganitrus additive (TB20 + R5) as the optimal formulation, achieving the highest Brake Thermal Efficiency and reduced Brake-Specific Fuel Consumption. Desirability analysis further confirmed the blend’s superior performance and emissions characteristics, with a desirability rating of 0.9282. This work highlights the potential of hybrid optimization approaches for improving biodiesel performance and emissions without engine modifications, contributing to the advancement of sustainable energy practices in internal combustion engines.

## Introduction

### Importance of Biodiesel

With rapidly increasing consumption rates, internal combustion engines (ICE) are expected to significantly accelerate the depletion of petroleum resources. When running an ICE on diesel fuel, there are two extra factors to consider: rising emissions of exhaust gases and the cost of crude oil^1^. Major polluting gases contributing to air pollution, like CO, unburned Hydro Carbon (UHC), and smoke opacity, affect human health^2^. Finding a sustainable alternative to fuel is essential to address these issues and halting climate change because of these and other problems^3^. One of these substitute fuels is biodiesel^4^, produced by transesterifying biomass or plant seeds. A renewable fuel without any petroleum is biodiesel^5^ and can be used in engines with or without modifications^2^. The percentage of biofuel used in the automobile sector is rising quickly in the twenty-first century due to environmental reasons, socio-economic factors, and foreign exchange^6^.

Both edible and non-edible vegetable oils are used as raw materials to create biodiesel^7,8^. Biodiesel made from four different vegetable oils was investigated by Rakopoulos et al.^9^. They tested the minibus engine’s performance using cotton seed, sunflower, corn, and olive oils. They reported that using these vegetable oils decreases smoke, a minor rise in NOx, and a very modest rise in the amount of UHC and CO released. In addition to investigating how to make biodiesel from canola oil, Roy et al.^10^ also examined the emissions produced by direct injection (DI) diesel engines (DE). The findings demonstrated that pure diesel fuel produced up to 5% more carbon emissions than pure canola oil. NOx emissions could go down or stay at diesel fuel-like levels. Adam et al.^11^ evaluated the enhanced waste source fuel’s combustion, efficiency, and internal combustion engine characteristics for exhaust emissions. Improved waste cooking oil produced 14% more power and 13.8% more torque than normal diesel. Fuel for disposing of waste plastic had the lowest NOx emissions due to its low combustion pressure curve. Diesel fuel was replaced with mixes of neem oil biodiesel.

In experiments conducted by Kannan et al.^12^. According to this study, CI engines using a B20 neem oil biodiesel blend and diesel achieved results that were quite similar concerning brake thermal efficiency (BTE)^13^. used propane additive with waste seed biodiesel and investigated its performance and emission features. They found improved performance features using the propane additives at different proportions. An algal biodiesel blend-fueled DI diesel engine’s performance, combustion, and emissions features were evaluated by Elkelawy et al.^14^, 50% conventional biodiesel and 50% algal biodiesel were utilized in their experiment. N-N-pentane was utilized to improve engine performance. The performance, emissions, and combustion of a CI engine were studied by Kodate et al.^15^ using biodiesel made from warmed Dhupa seed oil as an alternative fuel. According to the results of this study, preheating blends enhance fuel spray characteristics by lowering viscosity, which increases engine performance while lowering CO and HC emissions while marginally increasing NOx and CO_2_ emissions. In investigating the properties of cottonseed methyl ester blend-powered VCR engines with substantially reduced engine tail-pipe emissions at full load, Bora et al.^16^ concluded that a 15% cotton seed biodiesel mix had better brake thermal efficiency. However, they did discover that combinations of cotton seed biodiesel created more NOx than diesel. Ardabili et al.^17^examined the results of incorporating various quantities of diethyl ether into a 20% cottonseed biodiesel operating under different loading conditions. When using a ternary blend of cottonseed biodiesel, NOx, HC, and smoke emissions were reduced compared to the baseline blend. Devarajan et al.^18^ prepared biofuel from leather waste fat and gave valuable suggestions for reducing emissions.

When alcohols are oxidized, aldehydes like formaldehyde and acetaldehyde are created. Aldehyde emissions rise by 40% when only a 10% blend of ethanol is added to gasoline (as is typical in American E10 gasoline and abroad). Even so, some study findings suggest that reducing the Sulphur content of biofuel mixtures reduces the amounts of acetaldehyde. Aldehydes and other potentially dangerous aromatic chemicals are released while burning biodiesel but are not controlled by emission laws^19^. In this investigation, tests are carried out to analyze various parameters such as thermal efficiency, brake-specific fuel consumption, emissions of CO, CO_2_, HC, and NO gases in the exhaust, and also smoke density. The test results indicate that the blend fuel B20 for teak biodiesels can be used in diesel engines without any engine modifications. There is a close resemblance between blended biodiesel and diesel in terms of performance and emissions. All the performance and emission plots were plotted against Load in the entire research ranging between 3.4 kgf to 20.8 kgf.

### Machine learning and RSM

In recent decades, the RSM using the desirability technique has been widely used and accepted for scientific investigations. The RSM technique statistically designs, tracks, and evaluates trials by calculating the values in a mathematical model, assessing its viability, and visualizing the model’s response^20,21^. RSM has only been used by a small number of academics to analyze biodiesel emissions and performance^22^ to improve biodiesel production variables^23^ and to analyze the combustion of biodiesel^24–27^.

ML and DL techniques in modeling have also gained prominence recently, along with optimization techniques^28,29^. Joshi et al.^30^ forecasted and optimized the performance of biodiesel made from palm sesame oil using RSM and extreme learning machines (ELM). Regarding performance prediction, they saw that the ELM model performed noticeably better than the RSM model. Zandie et al.^31^ used a multi-input, multi-output ANN to predict diesel-biodiesel-gasoline mixtures’ engine performance and combustion characteristics. They found *R*^*2*^ nearly united, demonstrating the proposed network’s accuracy in predicting the desired characteristics. A summary of studies utilizing hybrid ML and RSM techniques for predicting and optimizing engine responses is shown in Table 1.

**Table 1:** Summary of studies utilizing ML and RSM techniques for predicting and optimizing engine responses.

Ref.	Techniques	Parameters	Biodiesel	Remarks
^32^	3.6 kW Kirloskar single cylinder, 4 stroke, water-cooled, DI engine	Performance and emission parameters	Palm biodiesel-ethanol	The mix displays the highest efficiency at 20% compared to other blends.
^33^	1-Cylinder, air-cooled, direct injection diesel engine	Engine load, biodiesel blend, and injection pressure	Safflower Oil	The engine’s ideal operating parameters were 1484.85 watts of engine load, 215.56 bar of injection pressure, and 25.79% biodiesel ratio.
^10^	1-Cylinder,4 S, DI diesel engine	Criteria for performance and emissions	Jojoba	PSO outperformed GA in optimizing the parameters.
^11^	Four-cylinder DI diesel engine.	The fuel blend’s percentage of biodiesel and the engine’s processing parameters	Safflower oil	Lower emission features using RSM.
^12^	KIA 1.3, 4-cylinder, 4 S, SI gasoline engine.	Performance variables and emissions	Ethanol–gasoline blends	As the bioethanol % rises, brake thermal and volumetric efficiency increased.
^34^	1-Cylinder water-cooled, DI, VCR diesel engine.	The compression ratio, injection pressure, and injection timing	AzadirachtaIndica (Neem)	The ideal engine performance characteristics were discovered using RSM.
^35^	ANFIS and RSM	EGT and all types of emissions.	Nano diesel blended fuels	The test results and the ANFIS predictions show a significant correlation.

Alternate energy sources, such as agricultural organic wastes, could decrease reliance on fossil fuels^36^. Among municipal solid garbage, the use of electricity from biodegradable waste has grown in popularity^37^. In this regard, the bio-oil produced from teak fruit seeds can be considered a good option. Various biodiesel blends have been used in engine applications^38^. Teak biodiesel with a Rudraksha component has been used as a green fuel despite the paucity of research on it^39^. The research that came before it makes it clear that not many studies employ RSM, particularly regarding a desirable strategy for assessing, forecasting, and enhancing biodiesel output and emission characteristics^40,41^. It’s also common knowledge that test engine trials are expensive and time-consuming. Therefore, the tedious and taxing procedure of performing several trials may be avoided by applying ML to the prediction^42,43^. Numerous authors have also used ML methods to forecast the properties under investigation, and research indicates that ML has great potential for resolving biodiesel development challenges^44^. Another difficulty that should be investigated in sustainability and energy is the coupling of experiment design with meta-heuristic algorithms.

This study addresses several key research gaps in optimizing engine performance using hybrid fuel blends. The first step in filling these gaps involves the application of RSM. This statistical technique systematically explores complex relationships between multiple input variables (fuel blend composition, engine settings, and operational parameters) and output responses (fuel efficiency, emissions, and engine power). By designing RSM experiments, the study efficiently maps out how different factors interact and identifies optimal conditions for the performance of hybrid fuel blends. To further validate the effectiveness of RSM, the results are compared with the performance of several machine learning (ML) algorithms, including Artificial Neural Networks (ANN), K-Nearest Neighbors (KNN), Extreme Gradient Boosting (XGB), and Random Trees (RT). This comparison allows for a comprehensive evaluation of RSM’s predictive accuracy and robustness compared to modern data-driven models, offering insights into the strengths and limitations of each approach in the context of hybrid fuel blend optimization.

To optimize engine characteristics for hybrid fuel blends, the study employs advanced hybrid optimization methods that combine the benefits of various optimization strategies. These include gradient-based optimization, which fine-tunes engine parameters efficiently, and evolutionary algorithms, which explore a wider range of possible solutions to avoid local optima and identify the globally optimal configuration. By applying these hybrid methods, the study achieves an optimal balance between competing performance metrics, such as improving combustion efficiency, reducing emissions, and enhancing fuel economy. The advanced optimization approach ensures that the engine characteristics are refined to meet environmental and performance standards while considering the practical constraints of real-world operations. Overall, this integrated approach, combining RSM, machine learning models, and hybrid optimization addresses existing gaps in the literature and provides a more comprehensive, efficient, and sustainable solution for optimizing engine performance with hybrid fuel blends.

## Materials and methods

**Fig. 1:**
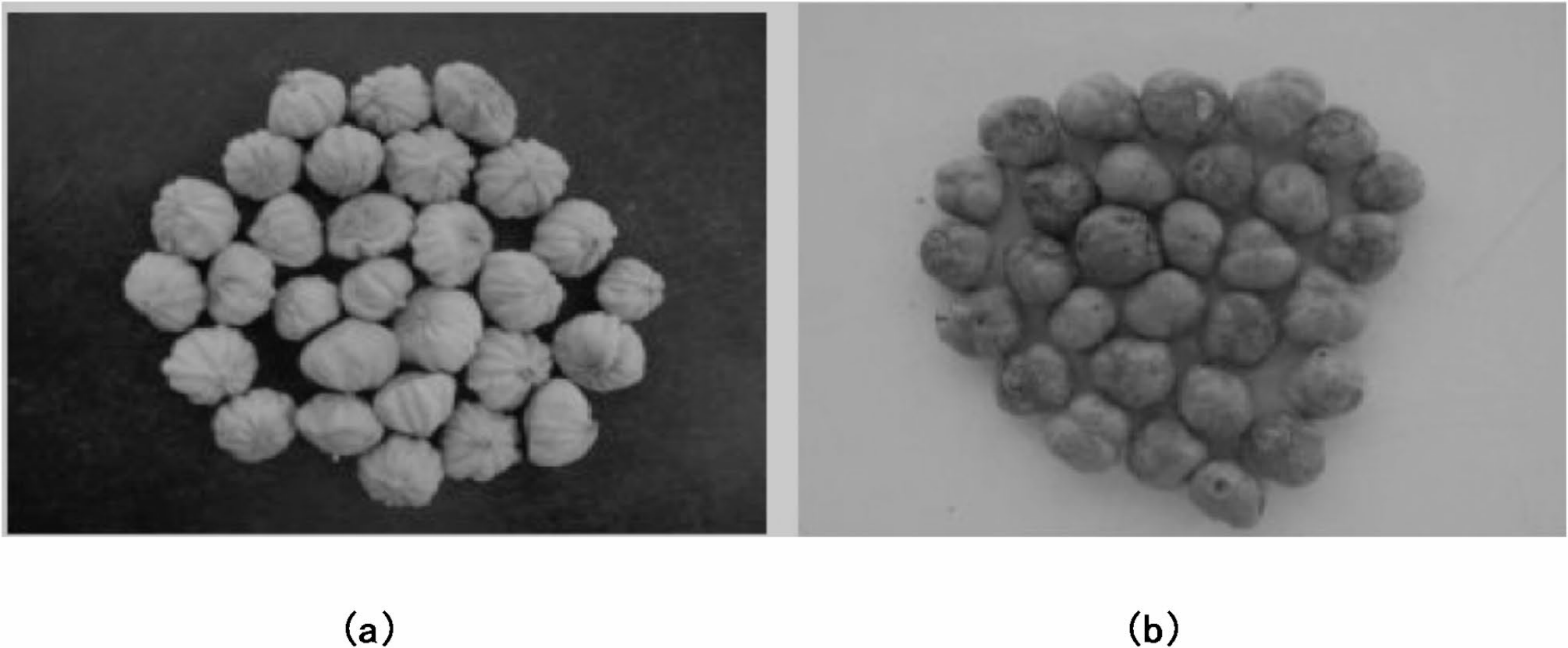
Fruits of teak: (**a**) young and (**b**) dried^2^.

Teak seed oil and Rudraksha seed oil were used to produce biodiesels. Water content and free fatty acids (FFAs) are the main characteristics of biodiesel production. A frequent and well-known chemical process called transesterification^22^ can be used when alcohol combines with triglycerides of fatty acids (vegetable oil) in the presence of a catalyst^39,45^. A 50:50 blend of Rudraksha and teak biodiesel was produced after turning the two oils into biodiesels, known as TR biodiesel. Tectona Grandis oil is more affordable and widely available than sunflower, soybean, and conventional oil^46^. Figure 1 depicts both fresh and dried fruits^47^.

Teak seed has a 41% oil content, rendering it appropriate for large biodiesel synthesis. The FFA level of less than 3% in biodiesel production has been readily transformed into biodiesel using a catalyst^48^. Similarly, the base catalyst transesterification procedure cannot reach the high amount FFA level in vegetable oils^49^. As a result, a 2 step trans-esterification procedure was used to convert elevated FFA content teak oils into biodiesel^6^. Transesterification processes were carried out for each oil sample, employing the appropriate quantity of methanol and KOH, cleaned with water, and warmed at 60–80 °C to eliminate residual water^50^. The transesterification reaction yielded a high percentage output of 79% biodiesel from teak oil^51^. Transesterification offers several advantages, such as producing high-quality biodiesel without pretreatment, avoiding soap formation, and a straightforward downstream purification procedure. In addition, the technique is non-toxic, renewable, biodegradable, and ecologically beneficial. It is safer because it has a higher flash point and fewer Sulphur compounds. It emits no carbon dioxide, 80% fewer hydrocarbons, and 50% fewer hazardous particles. It can be blended with regular diesel and does not contain petroleum residues. Compared to diesel, which is made from fossil fuels, it is less toxic.

A hydrometer assessed the fuel’s density in the test setting. A red-wood viscometer was used to determine the fuel’s viscosity. The bomb calorimeter device was used to determine the CV of each sample. Each sample’s fire point and flash point were determined using closed-cup equipment. Table 2 displays the physical characteristics of diesel, various biodiesels, PT biodiesel, and B20 with DEE.

**Table 2:** Diesel and biodiesels: Properties.

Fuels	Density in (kg m^− 3^)	Kinematic viscosity in (poise)	Calorific value in (kJ/kg)	Flashpoint in (°C)	Fire point in (°C)	Cetane number
Diesel	825	2.870	42,000	65	78	49
Teak Biodiesel	787	4.410	39,128	138	142	51
Rudraksha Biodiesel	820	2.86	37,826	65	75	51

EG rises in tropical and subtropical areas at prominences that reach 2000 m above sea level from the seashore. The Indo-Gangetic Plain and the Himalayan foothills are places where rudraksha is grown. Nepal, Indonesia, Java, Sumatra, and the mountainous areas of Burma and Burma are the native home of the rudraksha tree. The Rudraksha trees grow to great heights and bear tiny, fragrant, white flowers that blossom during the rainy season. These blossoms develop into berries that resemble blackberries and produce brownish-red seeds when they ripen. The tree is seasonal, meaning it grows all year long. EG is already sprouting in a subtropical region with 25–30 °C temperature ranges. After three years, these trees begin to bear fruit. Fruits start to appear in June and develop by August through October. The atmosphere and location of the EG tree, for example, determines the type of bead that forms. The climate in which the Himalayan beads develop gives them an appearance of being bigger, heavier, and more powerful^52^.

Rudraksha (EG) beads are made from a mixture of oxygen, hydrogen, nitrogen, carbon, and trace elements^30^. Without changing engines, EG biodiesel can be added to other biodiesels as an additive to increase efficiency and cut emissions while maintaining fair pricing. Table 3 shows the phytochemicals from the fruits of EG biodiesel.

**Table 3:** Phytochemicals from fruits of EG^51^.

Extract	Petroleum ether extract	Chloroform extract	Ethanol extract	Water extract
Phytochemicals	Phytosterol, fats, and fixed oil	Phytosterol	Alkaloids, flavanoids, carbohydrates, proteins and tannins	Protein, tannins and carbohydrates

## Test Setup

**Fig. 2:**
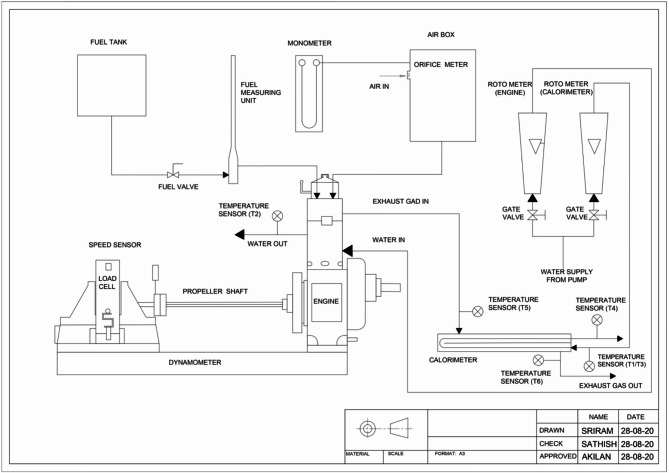
Experimental test setup^2^.

Utilizing a water-cooled, single-cylinder, four-stroke CI engine, biodiesel’s efficiency and emission properties were examined at 1500 rpm. The engine was rated for 5.2 kW and had a CR of 17.5:1. An eddy current dynamometer is coupled to a load cell, which provides the motor’s load. The Kirloskar TV-1 test engine’s schematic construction is seen in Fig. 2. In Table 4, its parameters are displayed. A data-gathering system tracks engine performance. The solenoid controller was used to measure a specific fuel consumption. An engine’s pulsation impact is measured by a surge tank, which also verifies that the intake manifold system keeps the airflow steady. The engine’s flywheel speed is measured via a non-contact sensor. The cooling water removes the heat emitted by the eddy current output. Monitoring also includes other aspects of combustion, such as pressure, heat transfer rate, and ignition delay. The engine load was applied using an eddy current dynamometer at different percentages: 20%, 40%, 60%, 80%, and 100% (maximum load).

**Table 4:** Engine specification.

Sl. No	Variable	Description
1	Engine	Kirloskar TV1, water-cooled, constant speed single cylinder, 4 S
2	Bore	87.5 mm
3	Stroke	110 mm
4	Compression ratio	17.5:1
5	Cylinder Volume	0.661 m^3^
6	Injection pressure	220 bar

### Uncertainty evaluation

Operational aspects of equipment calibration influence the risk of error or uncertainty during investigations, measuring instrument accuracy, human error, and experiment design. The number of runs was as per the design of the experiment rules. These rules will help carry out analysis using RSM and ANOVA techniques and optimize. Table 5 displays the findings from determining the degree of uncertainty for various parameters.

**Table 5:** Equipment list and related uncertainties^4^.

Equipment’s			
Parameters	Measuring technique	Accuracy	Uncertainty
Load	Strain gauge type load cell	± 10 N	± 0.2
Speed rpm	Magnetic pickup principle	± 10	± 0.1
Fuel flow measurement	Volumetric measurement	± 0.1 cm3	± 1
Temperature	Thermocouple	± 1 ◦C	± 0.15
Crank angle encoder	Magnetic pickup principle	± 1 deg	± 0.2
Pressure	Magnetic pickup principle	± 0.1 kg	± 0.1
Time	Stop watch (Manual)	± 0.1 s	± 0.2
Manometer	deflection Balancing of column of liquid	± 1 mm	± 1
CO	NDIR technique	± 0.02% volume	± 0.2
UHC	NDIR technique	± 10 ppm	± 0.1
NO	NDIR technique	± 12 ppm	± 0.3
Smoke	Opacimeter	± 1 HSU	± 1

To improve the reliability of our results, we have incorporated statistical tools such as confidence intervals and standard deviations to quantify the variability in the reported values, offering a more transparent representation of the data. Additionally, repeating experiments under controlled and identical conditions will help us validate the repeatability of the findings and reduce the impact of random errors.

Furthermore, we have enhanced the robustness of our predictive models by performing sensitivity analyses to identify key input parameters that contribute most significantly to variability. This approach will allow us to refine model inputs and improve prediction accuracy. By integrating these methodologies, we aim to strengthen the reliability of the results in Table 5 and provide a more comprehensive understanding of biodiesel blend performance and emissions optimization. We believe these efforts will enhance the overall scientific rigor of the study and effectively address the concerns regarding uncertainty analysis.

### Response surface method (RSM)

RSM assists in reducing the number of experiments, determining how input variables affect response variables^53^, and enhancing the response variables. The research’s input variables include the load (kW), biodiesel blends, and compression ratio (CR).

An equation must first describe the input and output relationship to use the response surface approach^54,55^. If a low-order polynomial function with accuracy might predict a link, the approximation function would be a first-order or linear model. (According to Eq. 1, the input and output responses are (A1, A 2., A k) and B, respectively^56,57^.

1 $$\:\text{B}={\text{f}}^{{\prime\:}}\left(\text{A}\right){\upbeta\:}+{\upepsilon\:}$$

Where $\:{\upepsilon\:}$ is the error. f(A) is a vector function with p elements that have powers $\:\left({\text{A}}_{1},{\text{A}}_{2}\dots\:,{\text{A}}_{\text{k}}\right)$ to a certain degree known as L (> 1). As a result, the equation for a first-order polynomial with L = 1 can be written as Eq. (2)^56,57^.

2 $$\:\text{B}={{\upbeta\:}}_{0}+\sum\:_{\text{i}=1}^{\text{k}}{{\upbeta\:}}_{\text{i}}{\text{A}}_{\text{i}}+{\upepsilon\:}$$

As an alternative, a higher degree polynomial is needed if the model includes a curve, a 2nd-order polynomial model shown in Eq. (3) below^56,57^.

3 $$\:\text{B}={{\upbeta\:}}_{0}+\sum\:_{\text{i}=1}^{\text{k}}{{\upbeta\:}}_{\text{i}}{\text{A}}_{\text{i}}+\sum\:\sum\:_{\text{i}<j}{{\upbeta\:}}_{\text{i}\text{j}}{\text{A}}_{\text{i}}{\text{A}}_{\text{j}}+\sum\:_{\text{i}=1}^{\text{k}}{{\upbeta\:}}_{\text{i}\text{i}}{\text{A}}_{\text{i}}^{2}+{\upepsilon\:}$$

Here, B represents the expected outcome, while I and j represent the linear and quadratic coefficients. In addition, k and $\:{\upepsilon\:}$ stand for the number of factors and an experiment’s random error, respectively, while $\:{\upbeta\:}$ is the regression coefficient.

### Desirability function approach (DFA)

One technique used in multi-objective decision-making and optimization is the Desirability Function Approach. It entails integrating several goals or answers into a single overall desirability function, which gives each response a desirability value according to predetermined standards or objectives^58,59^. These standards may consist of aiming for particular values for every answer, reducing or increasing them. The optimization and comparison of several replies at once are made possible by the desirability function, which converts individual response values into a single scale. This strategy is especially helpful when there are trade-offs between various solutions or competing objectives. The RSM of the desirability approach is used for input parameter optimization for output-measured values such as CO, NO_X_, BTE, UHC, BSFC, and smoke. The value of desirability ranges from 0 to 1. Des = 0 indicates a wholly unsatisfactory response, and des = 1 indicates a preferable response. Each response would aim to maximize, minimize, target, fall within a specific range, or be equal.

### Modelling using advanced machine learning (ML) algorithms

#### Artificial neural network

**Fig. 3:**
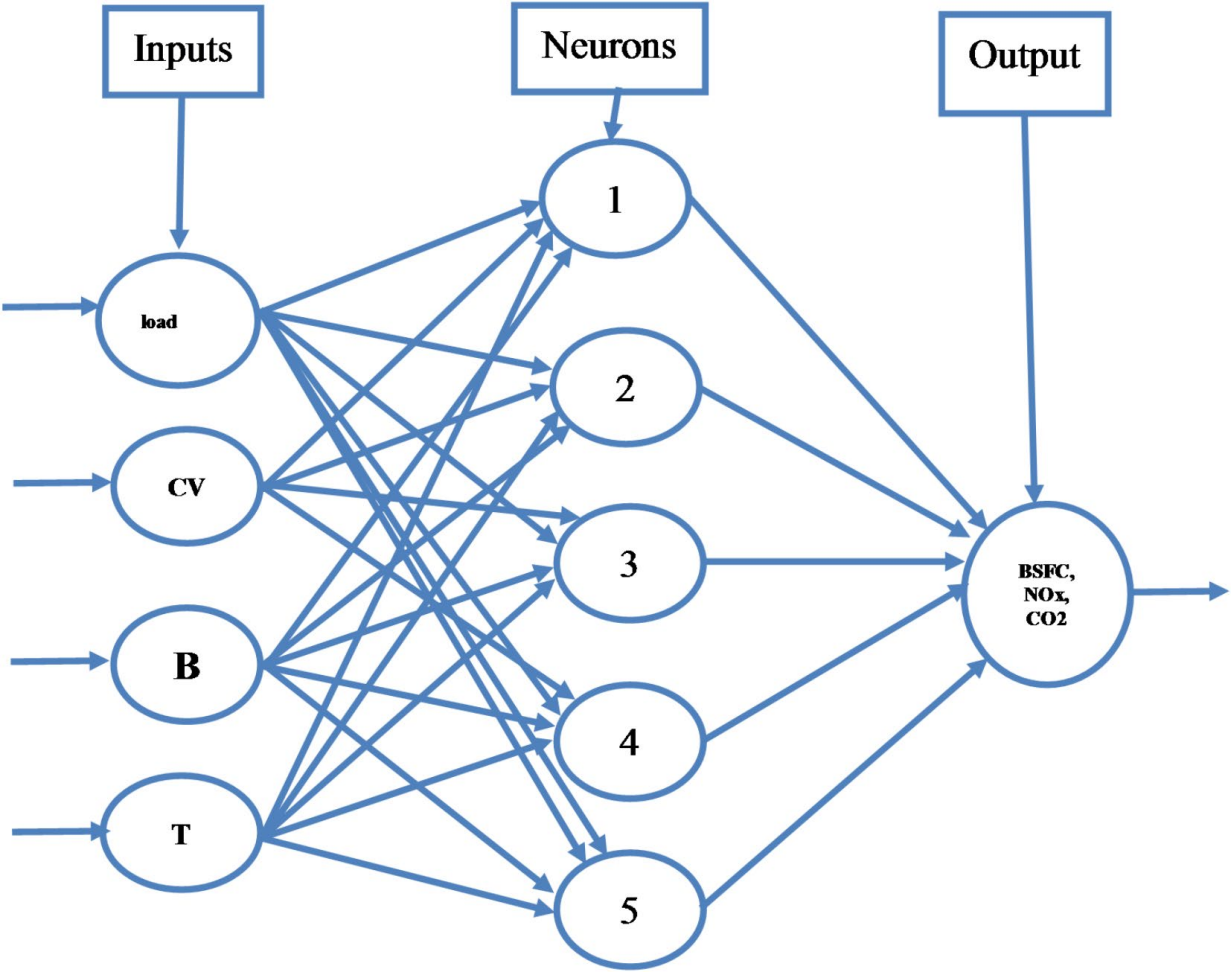
Schematic diagram of the neural network architecture. Input layer: Load (kg), CV-Calorific value (J/Kg), B-Blends, T-temperature in °C Output layer: BTE (%), BSFC (kg/kW-hr), NOx (PPM), Co_2_ (volume), HC (PPM).

ANN creates a sophisticated regression model using a network of interconnected neurons that can be used for prediction and decision-making issues^60,61^. ANN models successfully resolved difficult, non-linear issues for the ICE application^62,63^. However, picking the right network is essential for the accuracy of ANN models^64^. An ANN model comprises three layers: output, hidden, and input (Fig. 3). The input-output and input layers are first connected via the hidden layer^65^.

**Fig. 4:**
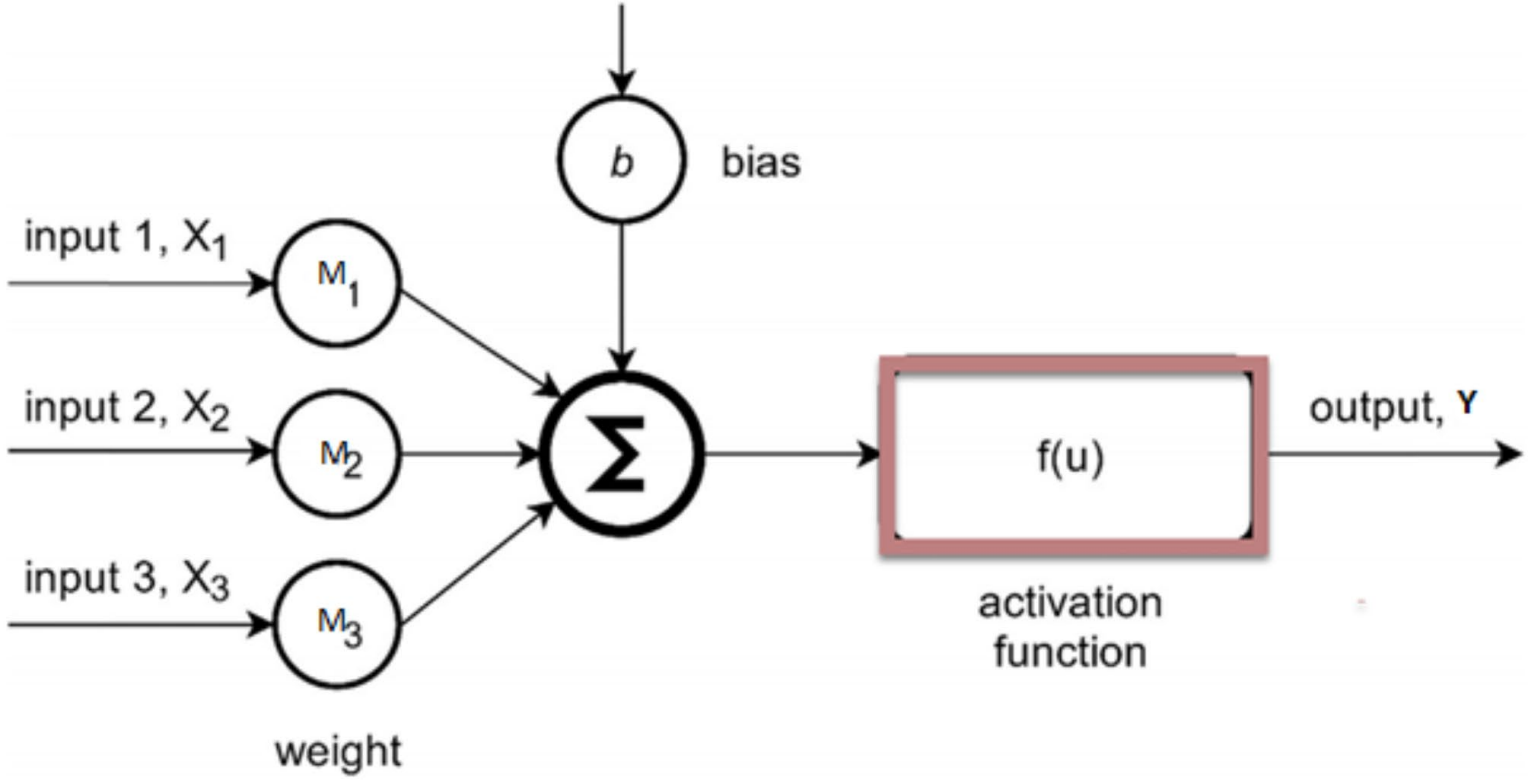
Structure of an artificial neural cell.

Additionally, it displays any associated weights with the input. As shown in Fig. 4, the weights are numerical representations of the node’s input strength. Consequently, the following can be used to sum up the model output^66^:

4 $$\:Y=t+\sum\:_{i=1}^{N}{m}_{i}{a}_{i}$$

#### Regression tree (RT)

A machine learning approach called RT is used for regression problems, the objective of which is to predict continuous numerical values. It is a member of the decision tree algorithm family. It works incredibly well when there is a complicated or non-linear connection between the goal variable and the input characteristics^67^. The RT algorithm operates by recursively dividing the feature space into smaller areas according to the values of the input features. At every node in the tree, a splitting criterion is utilized to determine which characteristic and threshold best divides the data into two groups^68^. This splitting process continues until a halting condition is satisfied, such as attaining a maximum depth, a minimum number of samples per leaf node, or a minimum variance reduction. The capacity of RT to capture non-linear correlations and interactions between characteristics is one of its main benefits, which makes it appropriate for datasets with intricate structures. Furthermore, RT is simple to understand since the resultant tree structure can be seen and understood, providing information about the factors that have the greatest bearing on the target variable’s prediction.

#### Gradient boosting regression algorithm

The boosting method is founded on the notion that, when judging a complex task, the outcomes of aggregating the judgments of several experts will be superior to those of a single expert^69^. The boosting method can increase the weak classification system’s accuracy based on the aforementioned traits. One type of boosting algorithm is the extreme gradient boosting algorithm. The mistake of the previous simple model is trained into each subsequent simple model^70^.

#### K-Nearest Neighbor (KNN) algorithm

The algorithm is used the most frequently in the ML toolkit. It obtains information by comparing an array given with matched training items. This approach’s effectiveness, simplicity, intuitiveness, and competitiveness allow it to handle classification and regression issues with up to significant dimensions. This approach uses the Euclidean distance equation to identify the k closest neighbors (Eq. 1) before choosing the primary class from among the k samples.

5 $$d\left( {\hat{p},\hat{q}} \right) = \sqrt {\left( {\hat{p}_{1} - \hat{q}_{1} } \right)^{2} + \left( {\hat{p}_{2} - \hat{q}_{2} } \right)^{2} + \cdots + \left( {\hat{p}_{n} - \hat{q}_{n} } \right)^{2} } = \sqrt {\mathop \sum \limits_{{i = 1}}^{n} \left( {\hat{p}_{i} - \hat{q}_{i} } \right)^{2} }$$

Where $\:d(\hat p,\hat q)$ defines the Euclidian distance, $\:\hat p$and $\:\hat q$are the data points consisting of *n* dimensions. This study’s greatest forecasting results were k values of 8 for NOx emissions and 7 for BTE.

### Model Limitations

Hybrid optimization approaches integrating RSM, and ANN has demonstrated significant potential for improving biodiesel performance and emissions characteristics. However, it is essential to recognize the limitations of these models to maintain a balanced perspective. While effective for experimental design and identifying optimal operating conditions, RSM struggles with capturing complex non-linear relationships between variables. This limitation may reduce its accuracy in systems like engine behavior optimization, where such non-linearities play a critical role. Additionally, RSM relies on predefined models, making it less adaptable to dynamic or evolving datasets, which can restrict its applicability in real-world scenarios.

Similarly, while ANN offers superior accuracy due to its ability to model non-linear relationships and handle complex datasets, it comes with its challenges. ANN models are computationally intensive, requiring significant processing power and time, particularly for large datasets or intricate network architectures. Furthermore, they are often considered “black-box” models due to their lack of interpretability, making it challenging to derive physical insights from the results. This opacity can be a drawback in engineering applications where understanding the underlying relationships is crucial. Despite these limitations, the integration of RSM with ANN and meta-heuristic optimization techniques has proven effective in this study, emphasizing the need for a judicious selection of models and methods based on the specific requirements and constraints of the application.

## Result and discussions

Using a blend of teak biodiesel and rudraksha additive, the performance and emission characteristics of the test engine are examined and contrasted with those of the diesel. 5 ml Rudraksha bio additive included in the Teak biodiesel mixture by the ratio of 10%, 20%, 30%, 40%, and 50% by volume, which are denoted by TB10 + R5, TB20 + R5, TB30 + R5, TB40 + R5, and TB50 + R5for enhancing the emissions and performance characteristics. Usin a 5-gas analyzer, the emissions of biodiesel blends, including CO, CO_2_, HC, and NOx, were discovered. The AVL 437 C Smoke Meter was used to calculate the percentage of smoke opacity.

### Performance features

Tables 6 and 7 display the ANOVA findings for the response surface quadratic models of BTE and NOx emissions. The results presented in Tables 6 and 7 are pivotal for understanding the robustness and reliability of the developed model. The significance level for the ANOVA analysis was set at 0.05, meaning that there is a 95% confidence level in determining the influence of the variables on the response^71^. A p-value less than 0.05 indicates that the corresponding factor or interaction has a statistically significant impact on the model^72^. The key findings are discussed:

- *Significant Interaction Coefficients*: The results indicate that interaction coefficients for BTE and Nox emissions are significant (*p* < 0.05). This signifies that changes in the independent variables (e.g., blend composition, engine parameters) affect these responses meaningfully. The significance of these coefficients validates that the model accurately captures the interactions between variables influencing both engine performance and emissions.
- *Effect on Model Accuracy*: The statistical significance of interaction coefficients enhances the overall predictive accuracy of the model. It demonstrates that the relationships between variables and their combined effects on BTE and NOx are not due to random variations but are consistent and reliable. This validation ensures the model’s applicability for optimizing engine responses in real-world scenarios.
- *Practical Implications*: The significant p-values emphasize the importance of selecting and controlling key variables to achieve desired engine performance and emissions outcomes. Optimizing the interaction between blend ratios and operating conditions could maximize BTE while minimizing NOx emissions.

By confirming the statistical significance of these interactions, the study reinforces the credibility of its findings, providing a strong foundation for practical applications in biodiesel optimization and engine performance enhancement.

The additional diagnostic criteria for assessing the created model of response variables are displayed in Tables 8 and 9, respectively. The R^2^ score for BTE is 0.9295. Its proximity to 1 shows how accuracy and adequacy of the model. There is a difference of less than 0.2 between the Adj. R^2^ value and the Pred. R^2^ value demonstrates their reasonable agreement^66^. The value of sufficient accuracy is 28.1190. This demonstrates the model’s accuracy.

**Table 6:** ANOVA table of BTE (%).

Source	Sum of Squares	df	Mean Square	F-value	*p*-value	
Model	2581.77	9	286.86	90.00	< 0.0001	Significant
A-LOAD	1549.43	1	1549.43	486.10	< 0.0001	
B-CV	4.42	1	4.42	1.39	0.2497	
C-Blends	6.64	1	6.64	2.08	0.1608	
AB	12.45	1	12.45	3.91	0.0488	
AC	6.09	1	6.09	1.91	0.1787	
BC	10.59	1	10.59	3.32	0.0498	
A^2^	325.08	1	325.08	101.99	< 0.0001	
B^2^	4.77	1	4.77	1.50	0.2321	
C^2^	4.82	1	4.82	1.51	0.2297	
Residual	82.87	26	3.19			
Cor Total	2664.65	35				

**Table 7:** ANOVA table of NOx (ppm).

Source	Sum of Squares	df	Mean Square	F-value	*p*-value	
Model	2.444E + 06	9	2.715E + 05	90.99	< 0.0001	Significant
A-LOAD	1.989E + 06	1	1.989E + 06	666.35	< 0.0001	
B-CV	3651.20	1	3651.20	1.22	0.2788	
C-Blends	864.63	1	864.63	0.2897	0.5950	
AB	12897.29	1	12897.29	4.32	0.0476	
AC	1947.44	1	1947.44	0.6526	0.4265	
BC	1356.03	1	1356.03	0.4544	0.5062	
A^2^	78525.16	1	78525.16	26.31	< 0.0001	
B^2^	2667.00	1	2667.00	0.8937	0.3532	
C^2^	20.76	1	20.76	0.0070	0.9342	
Residual	77590.12	26	2984.24			
Cor Total	2.522E + 06	35				

**Table 8:** Model evaluation (BTE (%)).

Std. Dev.	1.79	*R* ^2^	0.9295
Mean	28.69	Adjusted R^2^	0.9581
C.V. %	6.22	Predicted R^2^	0.9410
		Adeq Precision	28.1190

**Table 9:** Model evaluation (NOx (ppm)).

Std. Dev.	41.68	*R* ^2^	0.9855
Mean	805.00	Adjusted R^2^	0.9759
C.V. %	5.18	Predicted R^2^	0.9465
		Adeq Precision	38.7918

**Fig. 5:**
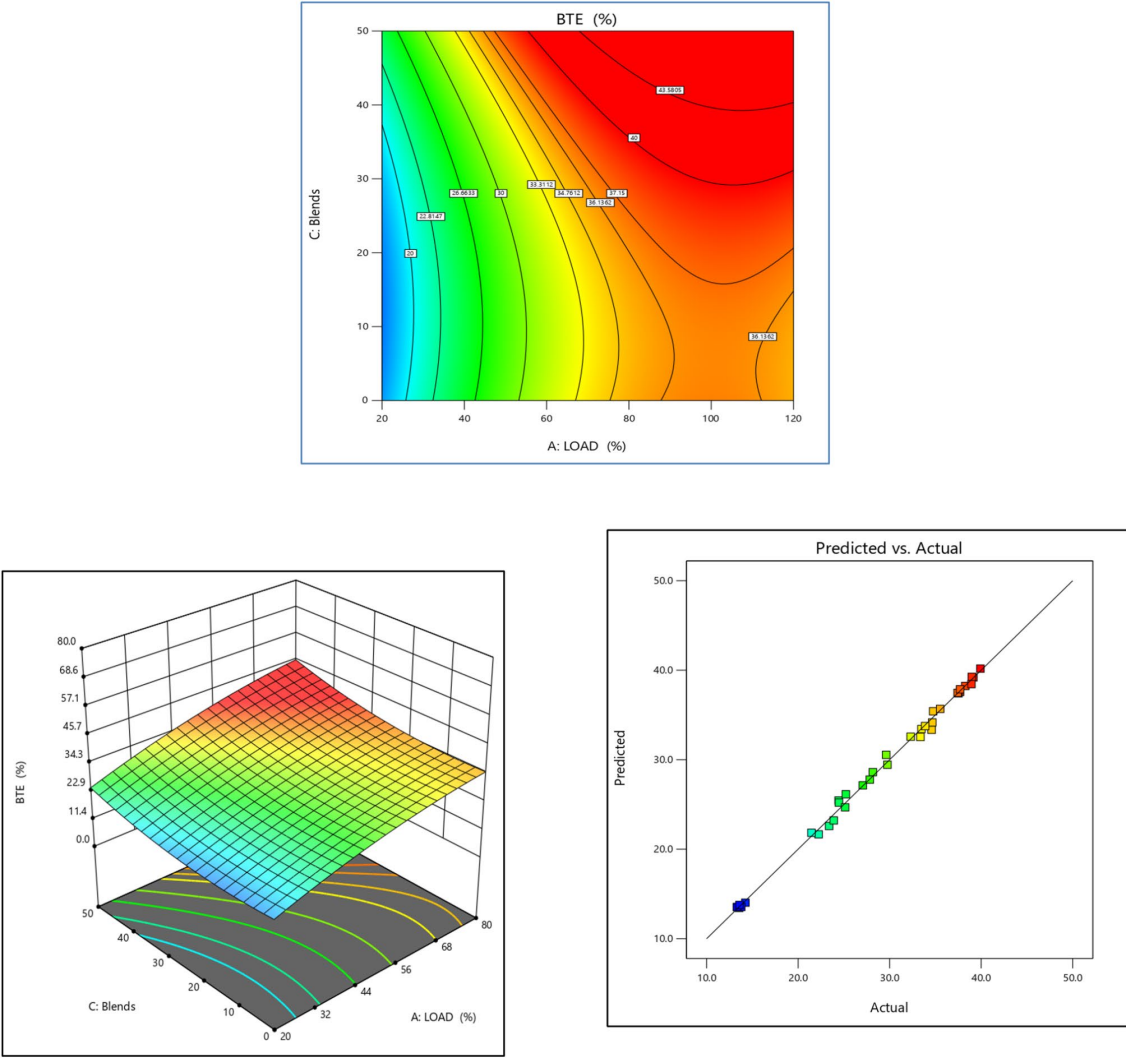
BTE (%) vs. Load for TB + R5 Blended Biodiesel (**a**) Contour plot (**b**) Surface plot (**c**) Actual vs. Predicted RSM graph.

A comparison of BTE for teak biodiesel with 5 ml EG additive in the test engine is displayed in Fig. 5. The BTE assesses the useful power output produced by the combustion of test fuels biodiesel^4^. Biodiesel has a lower heating value than diesel fuel, but it may still be converted into mechanical energy by an engine with an efficiency comparable to that of diesel fuel^73^.With comparison to the other test fuels, TB20 + R5 fuel had the maximum BTE under low load conditions^2^, most likely as a result of EG’s superior vaporization and mixing capabilities. The BTE was reported to be 26.41% for diesel and 31.52% for the TB20 + R5 blend at full load. Both diesel and biodiesel mix often experience a reduction in efficiency when the load exceeds the maximum. Figure 5c shows the prediction of the RSM model. The projected values are closer to the linear fit line, indicating that this model can correctly anticipate the engine output responses^74^.

**Fig. 6:**
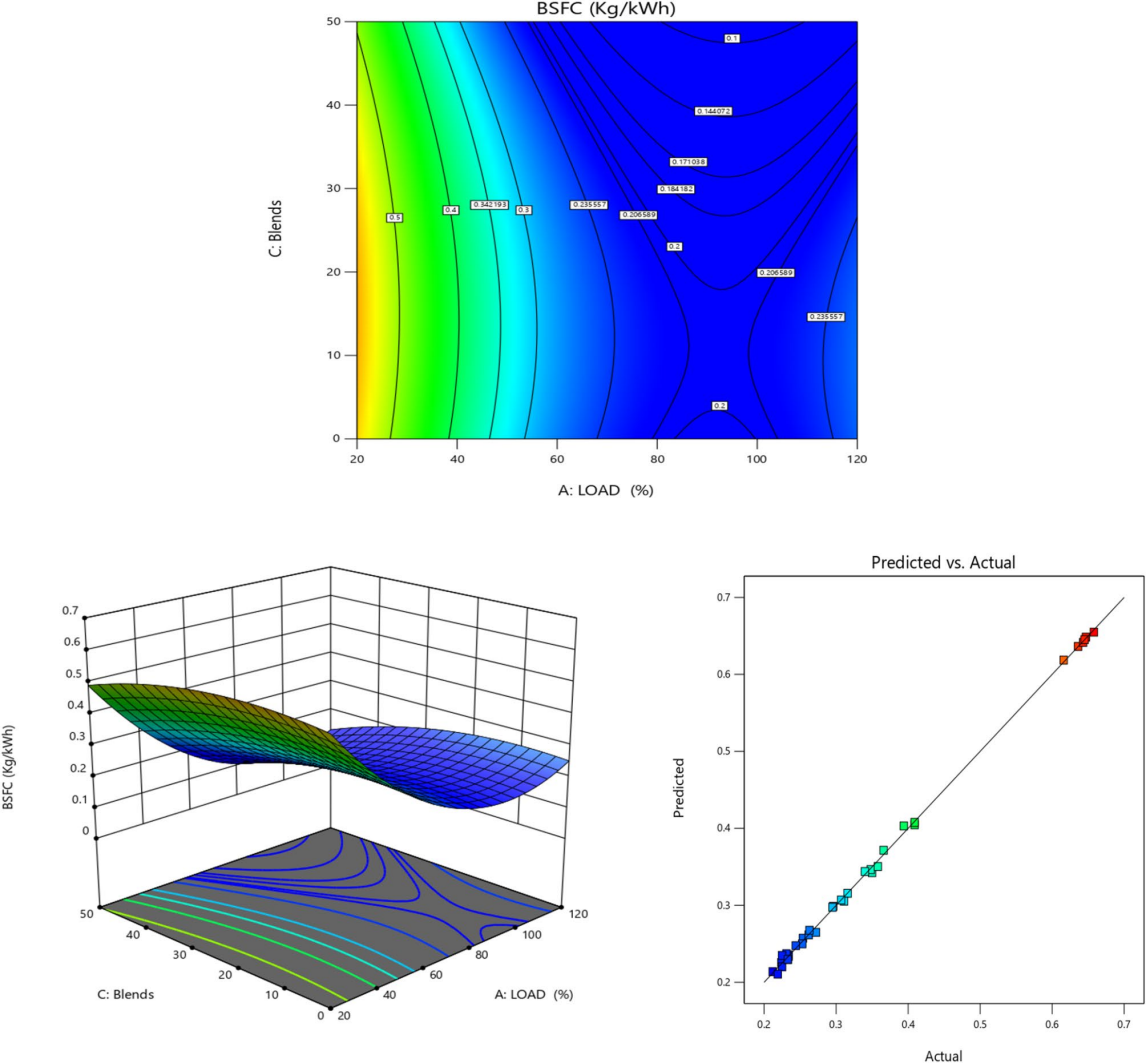
BSFC vs. Load for TB + R5 Blended Biodiesel (**a**) Contour plot (**b**) Surface plot (**c**) Actual vs. Predicted RSM graph.

The BSFC is compared with the load in Fig. 6. Since the BSFC factor gauges the engine’s charge efficiency, it must be carefully considered^75^. It demonstrates how well engine fuel generates work^76^. As the load increases, the BSFC values fall, as seen^23^.This phenomenon is caused by fuel consumption increasing more slowly with load than brake power does^56^. It is also obvious that biodiesel fuels have higher BSFC values than diesel fuels^57^. Because biodiesel has lower heating values than normal diesel, this increase results BSFC^77^. However, the BSFC for TB20 + R5 blend at full load was nearly identical to diesel fuel^78^.The BSFC climbs after the full load for diesel and biodiesel mixtures.

**Fig. 7:**
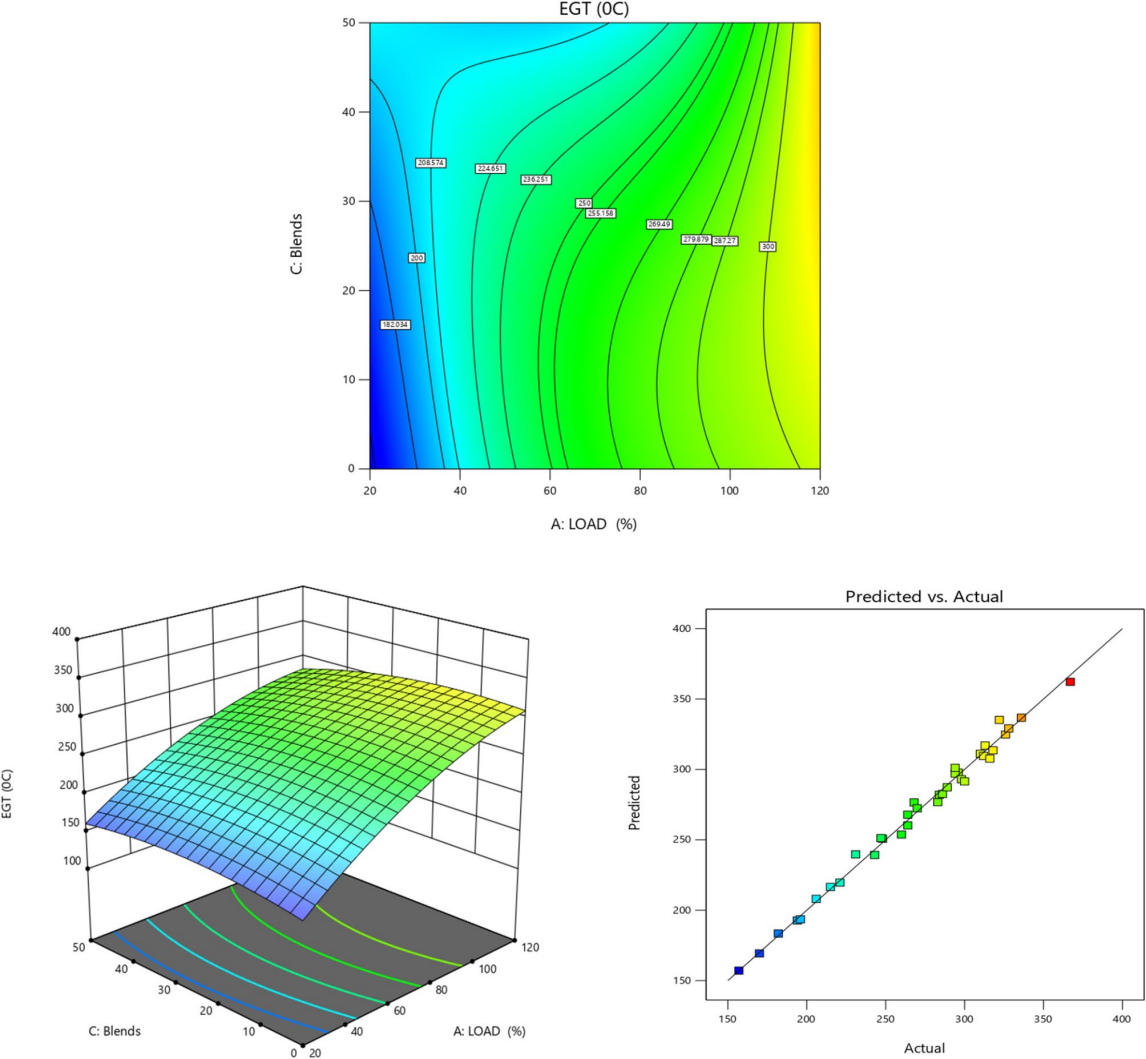
EGT (°C) vs. Load for TB + R5 Blended Biodiesel (**a**) Contour plot (**b**) Surface plot (**c**) Actual vs. Predicted RSM graph.

A comparison of EGT for the biodiesel utilized in the test engine is displayed in Fig. 7. Evaluation of the performance and combustion suggests that the EGT is a crucial factor during the test fuel’s combustion period^79^. The graph shows that the EGT of all fuels grew as the load increased^65,80^. These results indicate that when the load increases, the engine requires more fuel to maintain its output^8^. Also, it is noted that EGT for TB20 + R5 was lower than that of other higher percentage blends^81^. This is because the TB20 + R5 biodiesel has low viscosity and intrinsic oxygen (which favors the combustion).

### Emission features

**Fig. 8:**
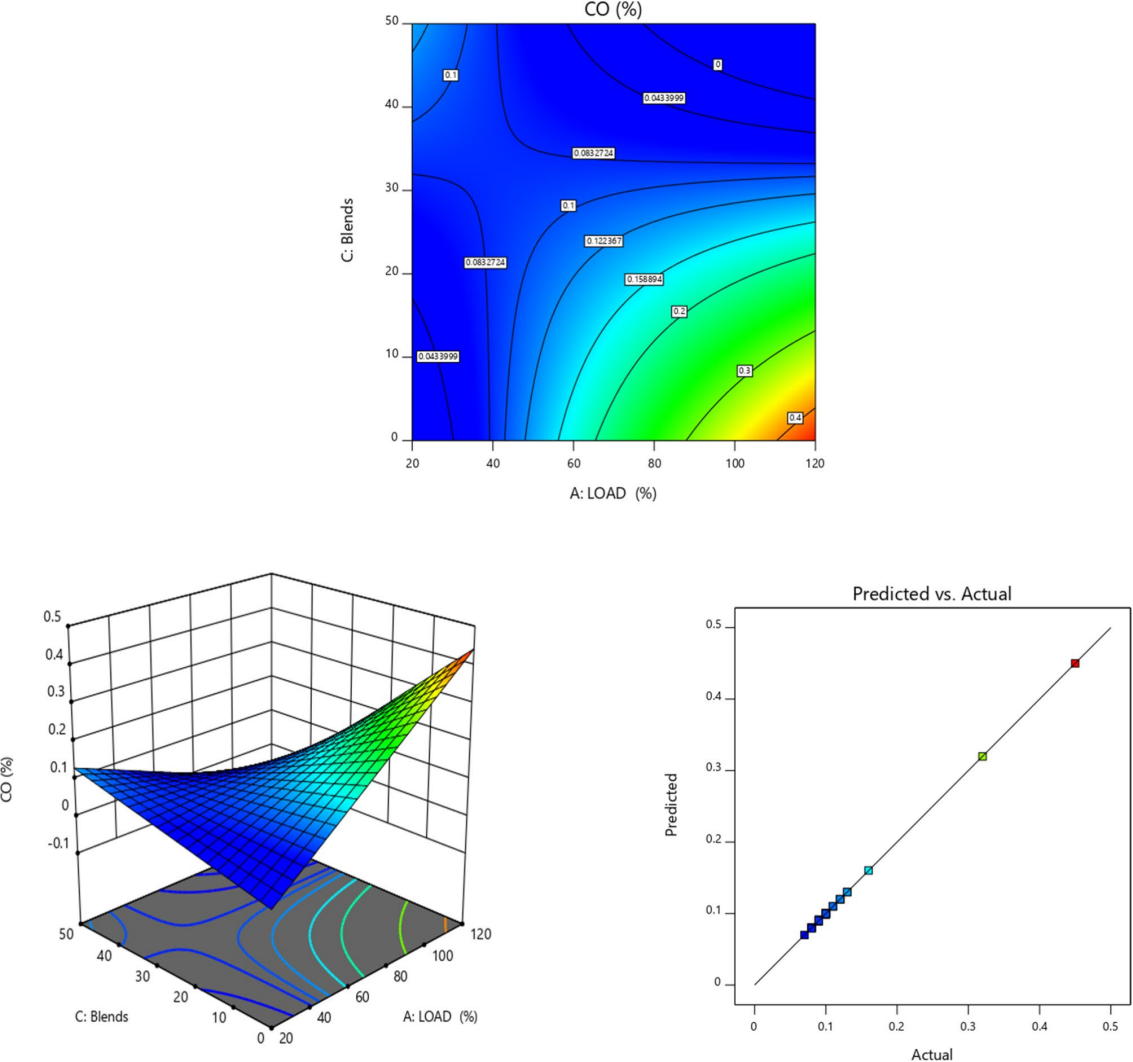
CO (%) vs. Load for TB + R5 Blended Biodiesel (**a**) Contour plot (**b**) Surface plot (**c**) Actual vs. Predicted RSM graph.

Figure 8 shows how CO emission varies depending on the load of various fuels. Comparing biodiesel and its lower concentration mixed with diesel, there is a lowering in the emission of CO^82^. The blend TB20 + R5 is reported to have the lowest CO emission levels^83^, after which a rise in CO emissions is noticed. This is because of the least viscous oxygen-enriched biodiesel^5^ due to the increased viscosity of biodiesel blends after TB20, the CO emission for biodiesel rises.

**Fig. 9:**
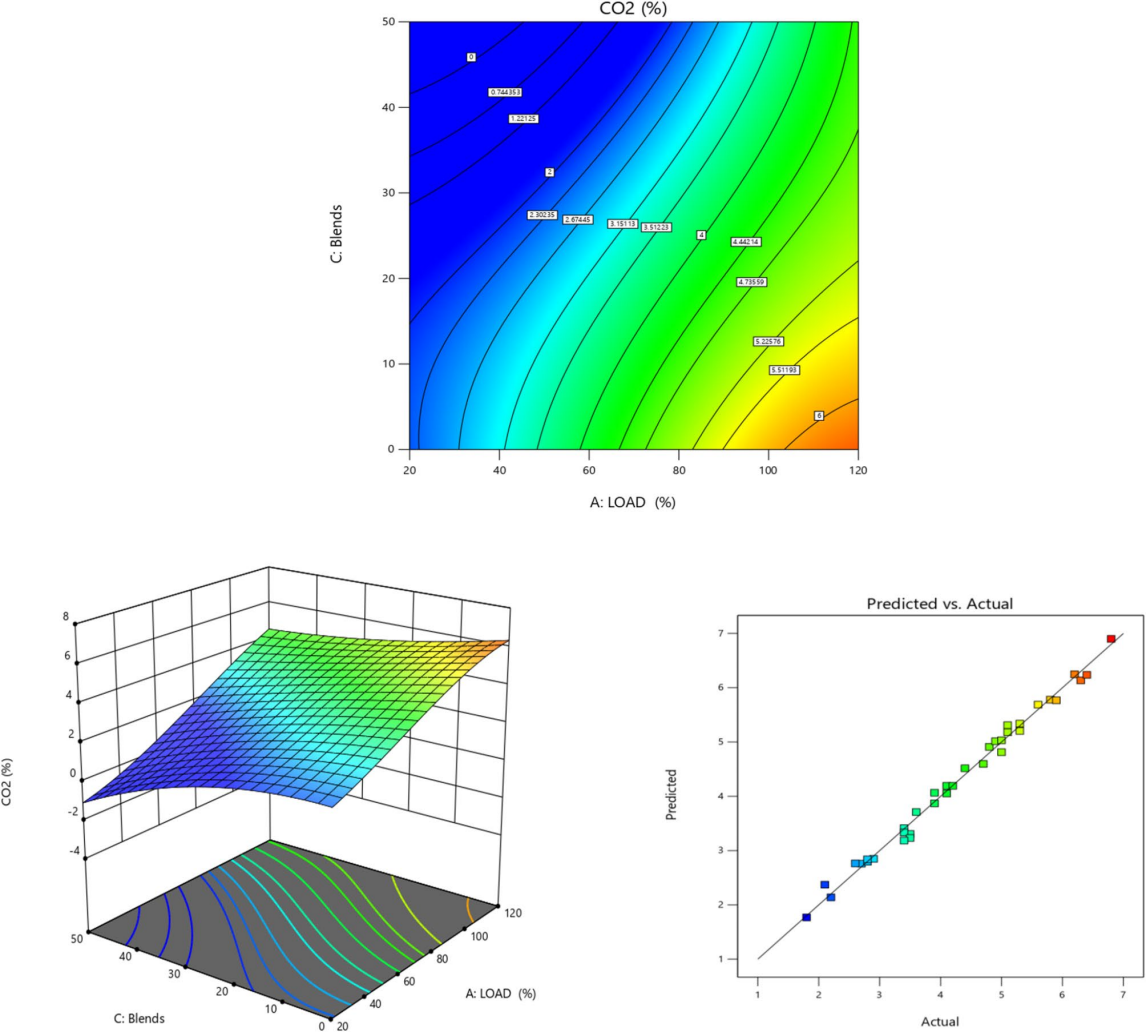
CO_2_ (%) vs. Load for TB + R5 Blended Biodiesel (**a**) Contour plot (**b**) Surface plot (**c**) Actual vs. Predicted RSM graph.

Figure 9 illustrates the test engine’s CO_2_ emissions when running on diesel and teak biodiesel blends under various loads. It has been noted that biodiesel blends with higher concentrations emit high CO_2_ due to incomplete combustion^84^. Meanwhile, for the same amount of fuel consumed, biodiesel with low concentration due to substantially lower carbon content oil^85^ and higher oxygen content emits lower CO_2_. The TB20 + R5 was noted for the lowest CO_2_ emissions under all load circumstances due to complete combustion^86^.

**Fig. 10:**
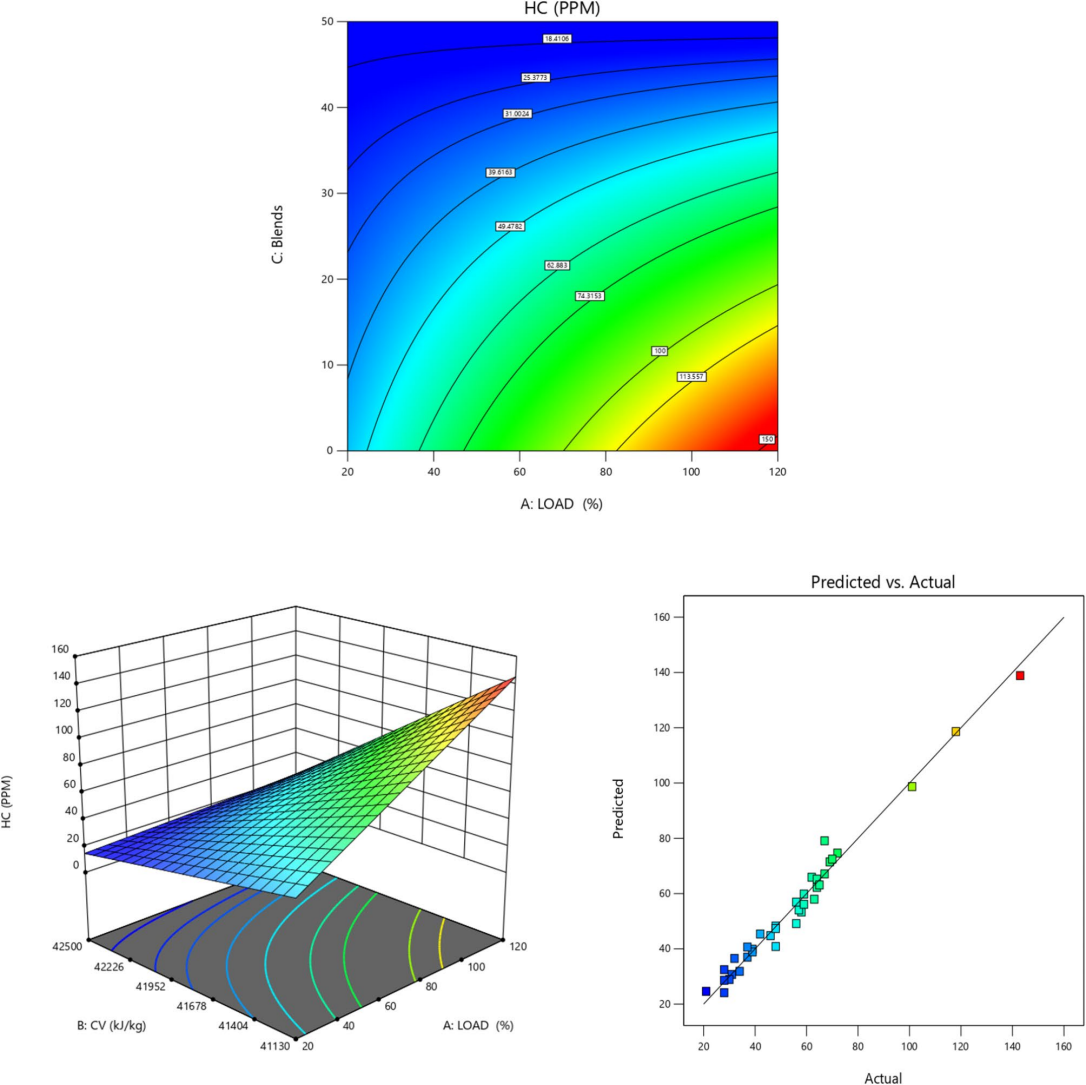
HC (ppm) vs. Load for TB + R5 Blended Biodiesel (**a**) Contour plot (**b**) Surface plot (**c**) Actual vs. Predicted RSM graph.

Figure 10 shows the disparity of HC emissions between load and biodiesel mixtures. Higher engine loads are observed to produce unusually high levels of HC emissions because more gasoline is pumped into the engine cylinder^87,88^. In atmospheric engines, the air is constant regardless of the load, preventing more fuel particles from burning^89^. At high loads, HC production results from an oxygen shortage^3,90^. The authors found a comparable reduction in HC emission by using TB10 + R5 and TB20 + R5 blends^18,91^ (as compared to other high-concentration blends) that demonstrates the potential for further HC reduction with coated engines.

**Fig. 11:**
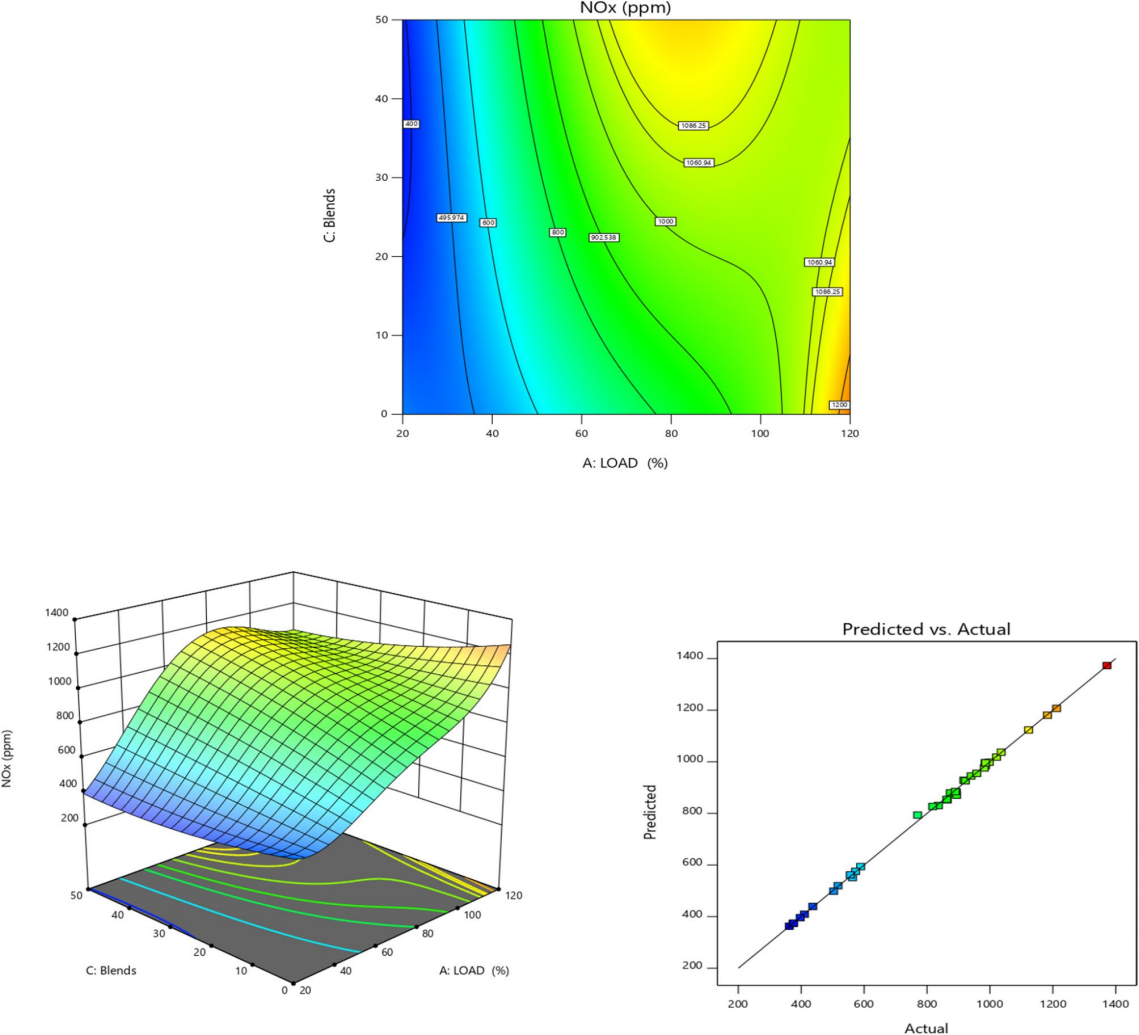
Load vs. Emission characteristics of NOx for TB + R5 Blended Biodiesel (**a**) Contour plot (**b**) Surface plot (**c**) Actual vs. Predicted RSM graph.

Figure 11 depicts the NOx emissions for diesel and its blends. For all the fuels, an increasing tendency is shown as the load increases^91^. Higher load situations lead to increased cylinder and EGT^92^. It has been shown that temperature affects NOx generation in IC engines. The graph also demonstrates that EG additive reduces NOx effectively^93,94^. This reduction is brought about by the additive’s enhanced latent heat of evaporation, which reduces the combustion temperature and further cools the combustion chamber.

**Fig. 12:**
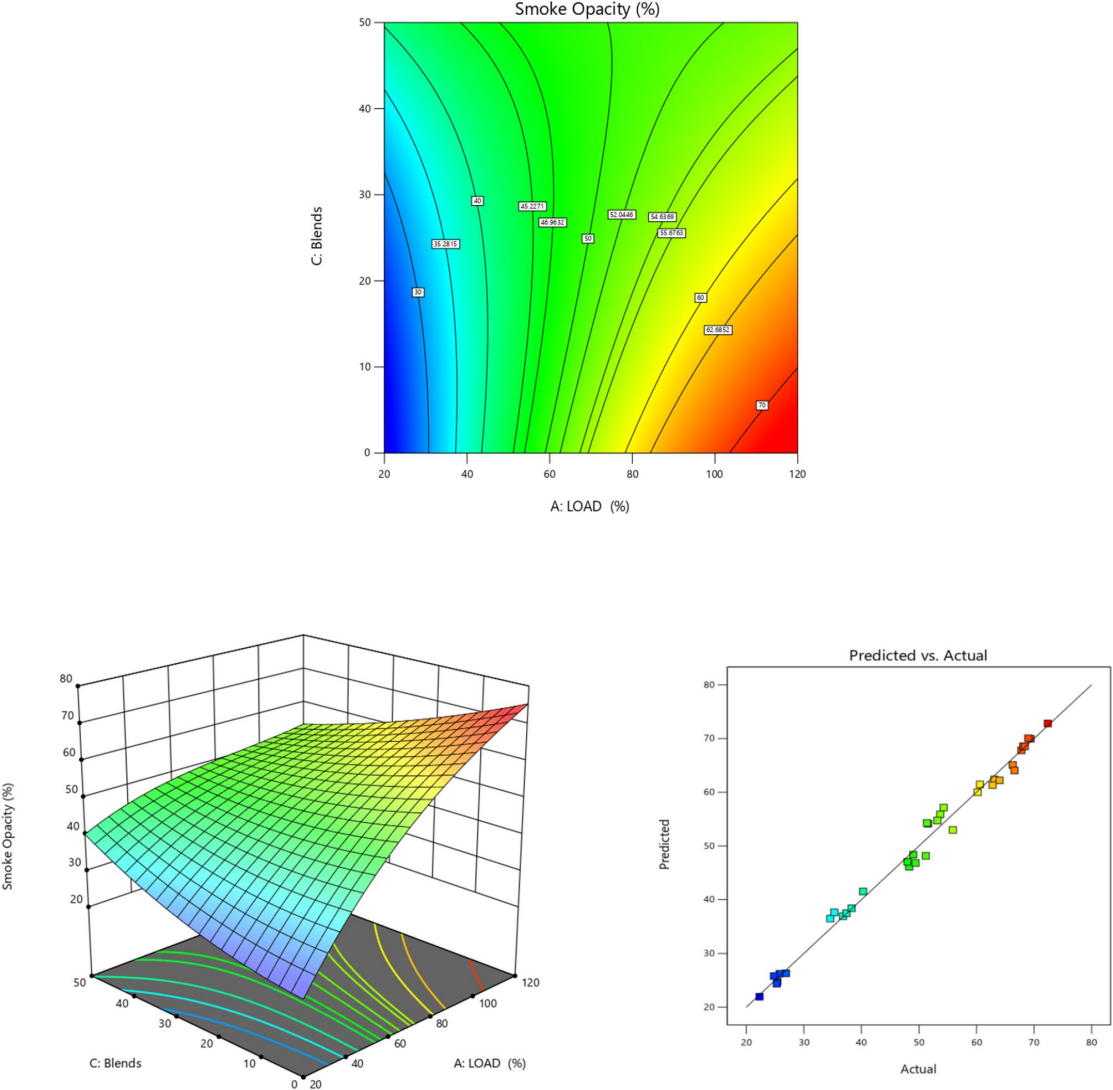
Load vs. Smoke Opacity for TB + R5 Blended Biodiesel (**a**) Contour plot (**b**) Surface plot (**c**) Actual vs. Predicted RSM graph.

The smoke level for various load circumstances utilizing various TB mixtures with EG additive is shown in Fig. 12. The smoke opacity of diesel engine exhaust determines the amount of smoke produced^95^. Other elements affecting smoke formation include viscosity, volatility, and quality. Figure 12 demonstrates that smoke opacity increases with increasing load for all fuels. Except for TB20 + R5 biodiesel^13^, which exhibits almost the same smoke opacity as diesel fuel^96^, all biodiesels have higher smoke opacities.

### Model predictability assessment

**Fig. 13:**
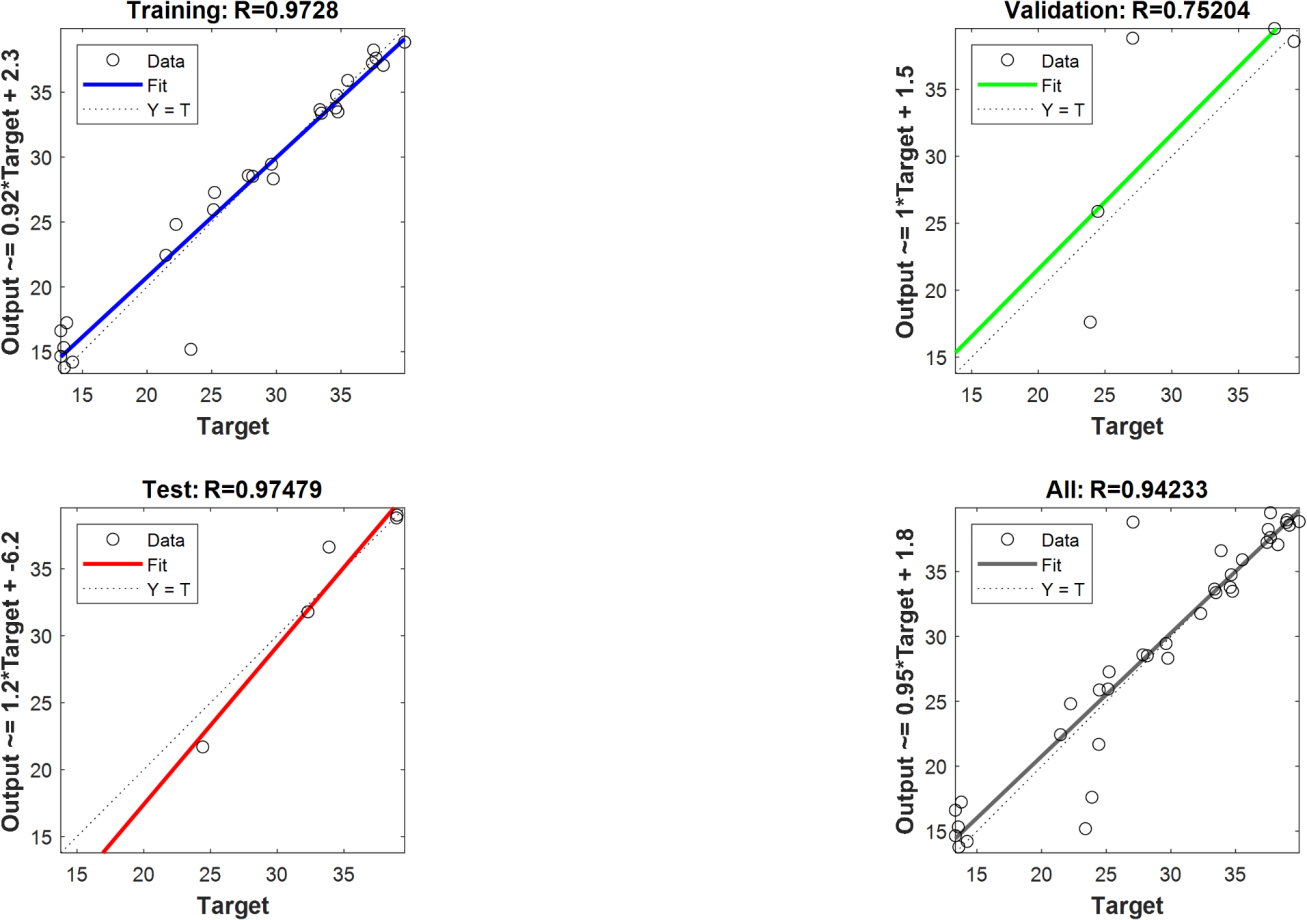
Optimum ANN regression performance at (**a**) 30 neurons in the BTE’s hidden layer (%).

**Fig. 14:**
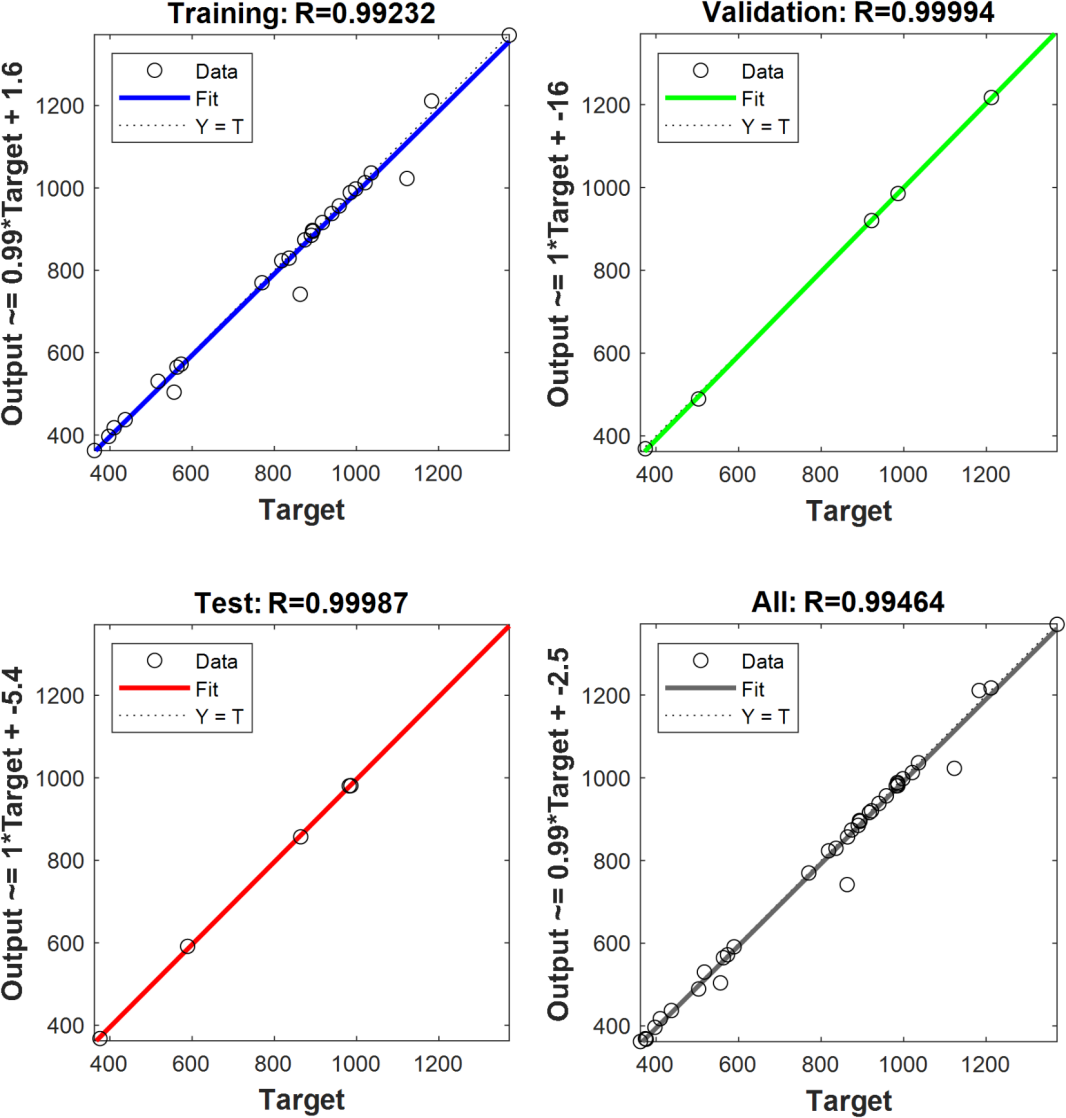
Optimum ANN regression performance at (**b**) forty neurons in a hidden layer for NOx oxides (ppm).

**Fig. 15:**
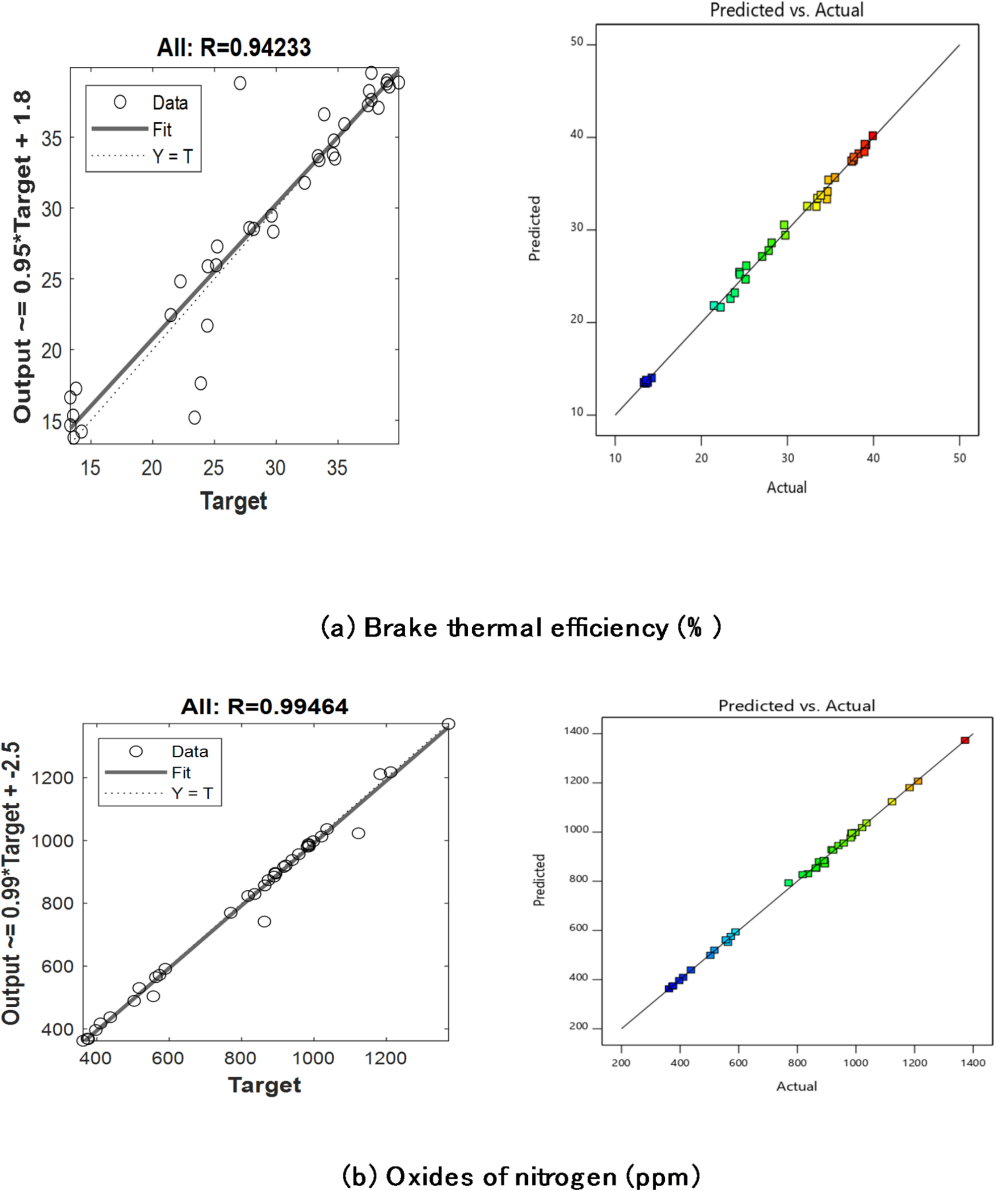
RSM and ANN estimation contrast.

The R^2^ correlation coefficient for validation data, preparation, and training of ANN models are displayed in Tables 10 and 11. The entire regression fit for the test, validation, and training sets is shown in Figs. 13 and 14. It demonstrates that the training, validation, test, and overall correlation coefficient values are close to 1, demonstrating the extremely accurate ANN model output responses^97^.The ANN and RSM models’ emission and performance parameters are compared, and both models can forecast the engine output responses because the anticipated values align with the linear fit line, as shown in Fig. 15. For ANN engine performance, the average R^2^ value was high(0.9423), as compared to RSM (0.9295)^98^. The ANN forecast matches experimental data more closely than the RSM prediction when comparing the R^2^ values of the ANN and RSM models^99^.

**Table 10:** Results of ANN for predicting BTE.

hidden layer in neurons	MSE
	All data
15	0.9220
20	0.9353
25	0.9413
**30**	**0.9423**
35	0.9352

**Table 11:** Results of ANN for predicting NOx emissions.

Number of hidden layer neurons	MSE
	All data
10	0.9916
15	0.9923
20	0.9932
30	0.9938
**40**	**0.9946**
45	0.9443

**Fig. 16:**
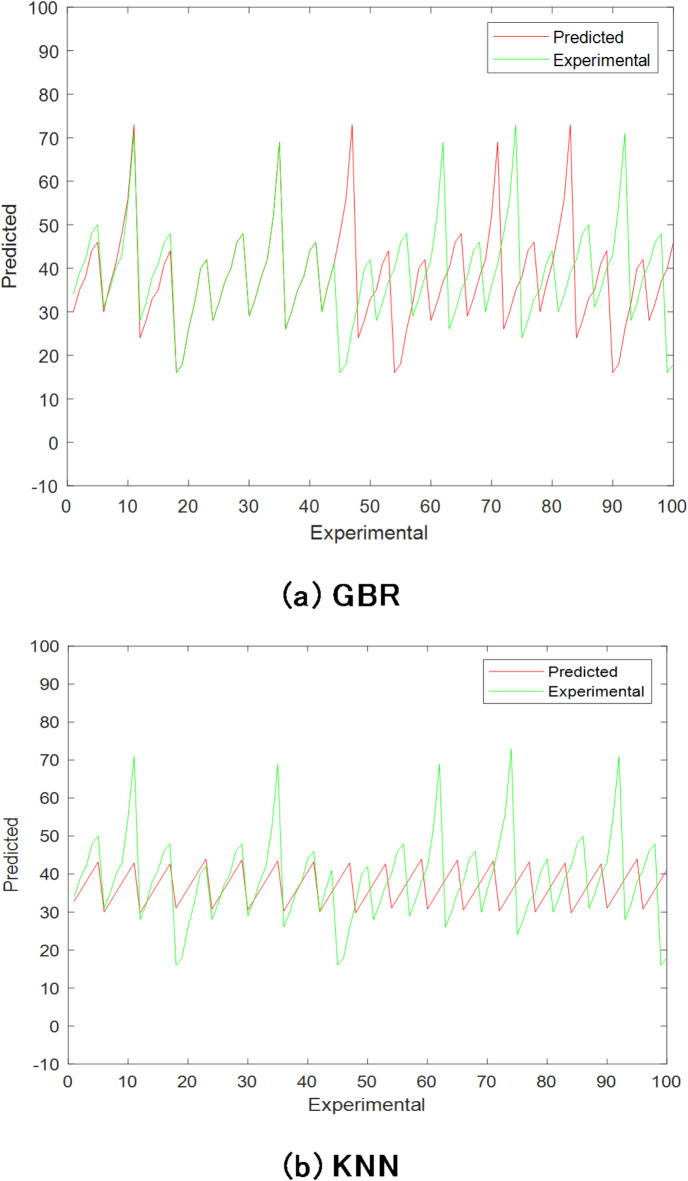
BTE prediction using machine learning (ML) techniques.

Forecasts for BTE (%) and NOx were also made using various ML regression models. Tables 12 and 13 present the regression model coefficients for forecasting *BTE (%)* and NOx, respectively. These models all have R^2^ values that were slightly lower than ANN, as can be seen. Figure 16 displays the BTE (%) forecast using machine learning approaches.

**Table 12:** Modelling coefficient for various BTE anticipating regression models.

Regression Model	MAE	MSE	RMSE	R^2^
RT	97.7371	29870.8045	151.6359	0.9143
GBR	118.7559	38144.2433	167.6888	0.9595
KNN	177.7836	70919.4550	241.6641	0.9233

**Table 13:** Modelling coefficient for various NOx anticipating regression models.

Regression Model	MAE	MSE	RMSE	R^2^
RT	141.7291	41410.3333	188.4311	0.9133
GBR	126.4091	45670.6565	182.0164	0.9402
KNN	134.5624	56031.3501	200.4754	0.9236

MSE stands for mean squared error, MAE stands for mean absolute error, and R^2^ stands for coefficient of determination. RMSE for relative root mean squared error.

### PSO and multi-objective dragonfly optimization (MODA) optimization for efficiency and emissions

The optimized values of outputs (*BTE (%)* and NOx (ppm)) using PSO are displayed in Tables 14 and 15. The optimization utilized the regression equations created with RSM as the goal function. Hence, the optimization is called Hybrid RSM-PSO and Hybrid RSM-MODA optimization. Load-80%, a 20% biodiesel blend, and 41,483 kJ/kg Calorific Value were the input variables for the maximum *BTE* (%). Thermal efficiency rises with increasing load^100^, and for mixtures containing 20% biodiesel, this appears to be scientifically meaningful^100,101^. The lowest NOx emission is observed to be 432 PPM for a blend percentage of 40 and the lowest blend of 10% (converged after 400 iterations).

**Fig. 17:**
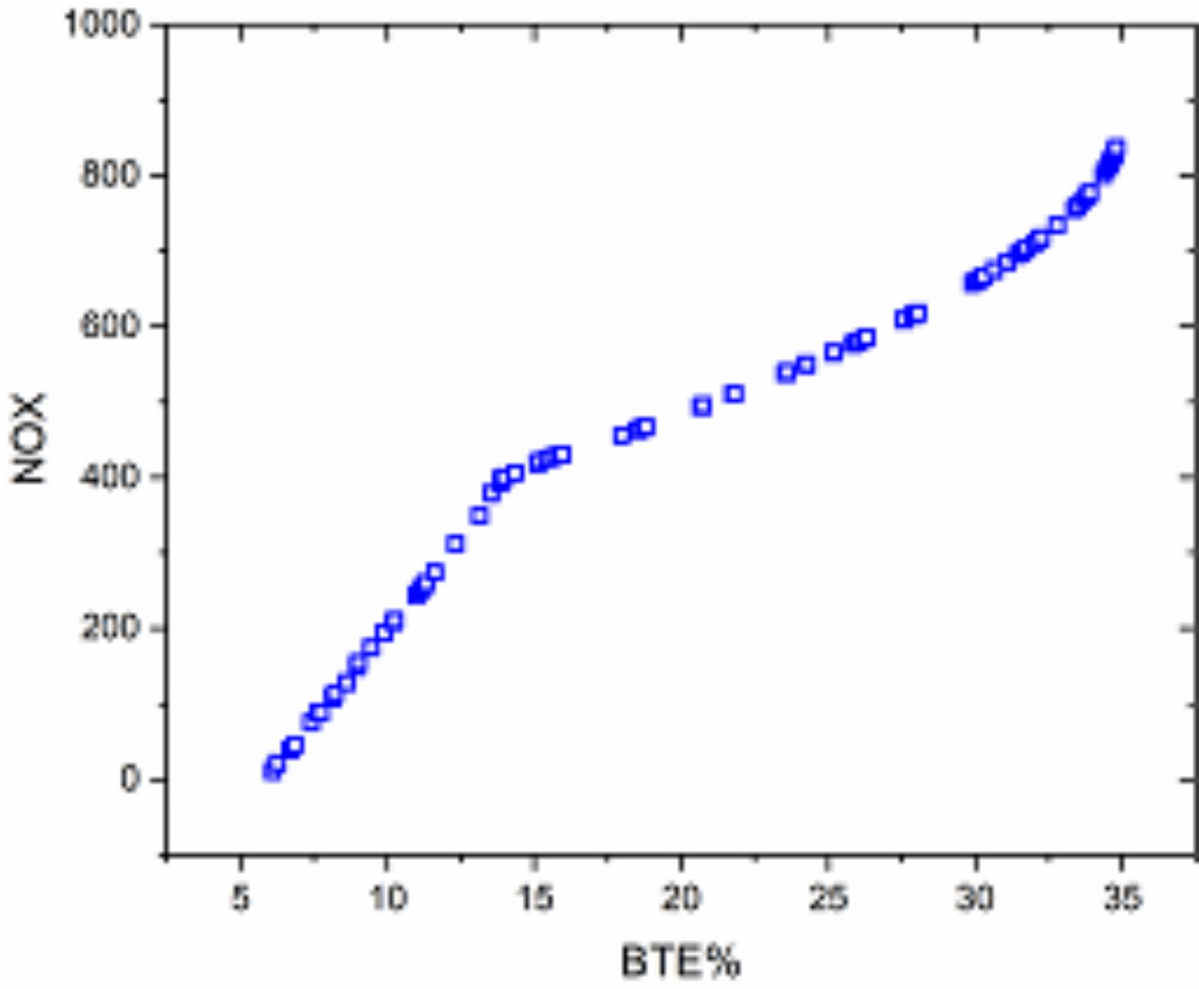
Pareto plot using MODA algorithm.

MODA^102^ carried out multi-objective optimization to increase engine efficiency while lowering emissions. Figure 17 displays the Pareto plot versus both of the target functions. Increasing BTE results in increasing NOx emissions. Low emissions are thus achieved with low loads and low biodiesel blends for additional input variables.

**Table 14:** The parameter’s ideal values for the greatest BTE (%).

Parameters	Value	*BTE* (%) = 39%
Load (%)	68	
CV	41,483	
Blends	20	

**Table 15:** The parameter’s ideal values for minimizing NOx (ppm).

Parameters	Value	*NO (ppm) =* 432
Load (%)	60	
CV	41,884	
Blends	10	

### Optimization using DFA

**Fig. 18:**
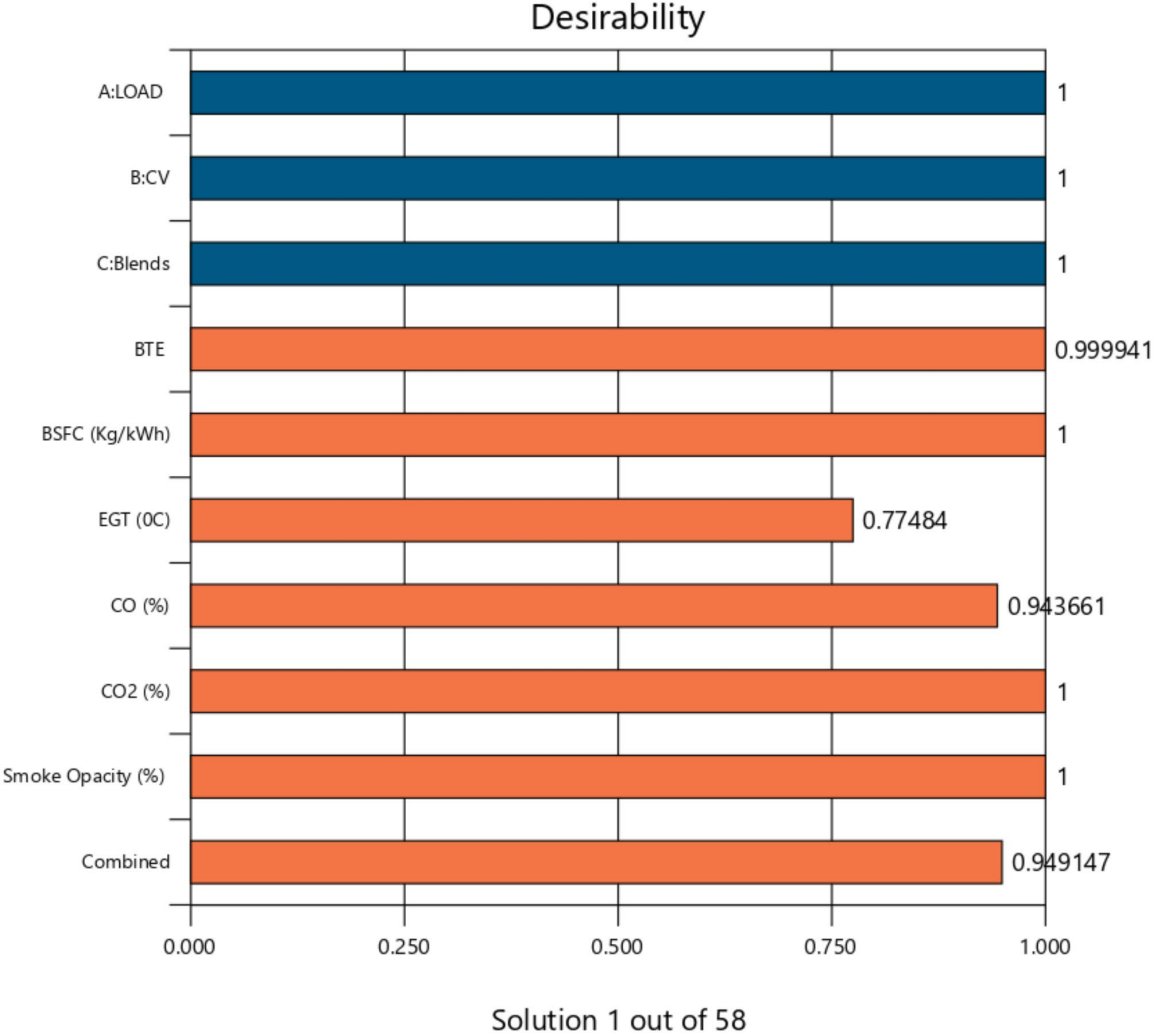
The desirability of all constraints.

The various engine input parameters are optimized by the RSM model^103,104^. The optimization setup is shown in Table 16. It consists of an objective, weights with lower and upper bounds, and importance (In the context of the DFA optimization, “weights” refer to the relative importance or priority assigned to each response variable when calculating the composite desirability function. These weights influence how much each response contributes to the optimization process). Blend, engine load, and compression ratio are three variables that kept it inside the range^8^. The remaining output variables (NOx, HC, BSFC, CO, EGT, CO_2_, and Smoke opacity) are minimized, and only BTE is maximized. DFA’s suitability can be shown by the combined desirability (D) and individual (di)^105^, which are both closer to one. Figure 18 displays a bar graph illustrating the desirability of each constraint for the best post-optimization solution. With a desirability score of 0.9282, the mixture TB20 with a 68 kg load is considered to have the best performance and emissions.

**Table 16:** Optimization setup for DFA.

Name	Goal	Lower Limit	Upper Limit	Lower Weight	Upper Weight	Importance
A: LOAD	is in range	20	120	1	1	3
B: CR	is in range	16.5	17.5	1	1	3
C: Blends	is in range	0	50	1	1	3
BTE	maximize	13.33	39.92	1	1	3
BSFC (Kg/kwh)	minimize	0.212	0.658	1	1	3
EGT (0 C)	is in range	157	367	1	1	3
CO (%)	minimize	0.07	0.45	1	1	3
CO_2_ (%)	minimize	1.8	6.8	1	1	3
NOx	minimize	362	1372	1	1	3

The desirability score of 0.9282, as shown in Fig. 18, highlights the efficacy of the optimization process, confirming that the TB20 + R5 blend, under the specified engine load of 68 kg, achieves an optimal balance between performance and emissions. This high desirability score indicates that the optimization objectives, such as maximizing BTE while minimizing emissions like NOx and Smoke Opacity, were met with high precision. Linking this to the desirability bar graph underscores the robustness of the DFA in achieving multi-objective optimization.

The MODA Pareto plot, presented in Fig. 17, complements the desirability analysis by visually representing the trade-offs between conflicting objectives, such as increasing BTE and reducing NOx emissions. The plot demonstrates that higher engine loads and biodiesel blends enhance BTE but at the cost of elevated NOx emissions, emphasizing the need for a compromise to achieve balanced outcomes. This trade-off is effectively managed using RSM-based multi-objective optimization and DFA, where weights and importance values guide the prioritization of objectives. Incorporating these linkages into the discussion provides a more explicit narrative connecting optimization outcomes, such as the desirability scores and MODA insights, to the practical implications of using TB20 + R5. This reinforces the study’s contribution to advancing biodiesel optimization strategies for sustainable energy solutions.

## Conclusions

A combination of TB biodiesel and an EG additive was tested experimentally in this study in line with DOE rules to evaluate its emission and performance traits. Particle swarm and dragonfly algorithms and the RSM approach were used to optimize and obtain the parameter’s optimum values. ML techniques were used to anticipate these properties. The result is described as follows:

- The blend of diesel and TB biodiesel with an EG addition showed an increase in BTE and a downward trend for BSFC with the load. TB20 + R5 blend showed maximum BTE under full load conditions. The BTE was reported to be 26.41% for diesel, and 31.52% for the TB20 + R5 blend at full load.
- The EG additive reduces NOx effectively. The use of this additive also resulted in less smoke, which was observed. As a result of its improved emission and performance characteristics, EG additives can be used as a substitute fuel without requiring modifications to the engine.
- The ANN model outperforms RSM results in terms of accuracy when the R^2^ readings of the two models are compared.
- For ANN engine performance, the average R^2^ value was high (0.9423) compared to RSM (0.9295). As a result, it was revealed that the ANN was generally more effective than RSM at estimating how various factors will impact the CI engines’ emissions and efficiency characteristics.
- With a desired score of 0.9282, the blend TB20 + R5 with 68 kg load is considered to have the best performance and emissions.
- A good match between the RSM models and the real data is suggested by the RSM technique’s accurate prediction of values that are similar to those found in the experimental data, which qualifies the models for parameter optimization. The desirability technique successfully attained optimal parameter values for the constructed RSM model.
- Single objective (PSO) and multi-objective algorithms (MODA) were applied to determine the parameter’s ideal values. TB20 + 5 ml biodiesel blend at its maximum load gave the maximum value of BTE (%) with lower NOx emissions according to MODA results.
- All ML algorithms gave R^2^ values very close to unity, demonstrating the suggested good network’s accuracy in predicting anticipated performance and emission characteristics.

### Future scope

Although there are few applications of AI to biodiesel systems, research indicates that it has a great deal of potential for removing barriers to biofuel development. Combining advanced machine learning techniques with neural networks may be possible to deliver significant intelligence for enhanced forecast models. Advanced hybrid optimization with AI can be used to find the best engine settings.

Future research could explore testing other biodiesel blends with varying feedstocks and additive compositions to expand the applicability of AI-driven solutions. Integrating real-time optimization systems that utilize AI and sensor feedback could enhance engine adaptability, allowing dynamic adjustments to operating conditions for sustained efficiency and reduced emissions. These advancements would push the boundaries of biodiesel technology and promote its widespread adoption.

## Data Availability

All data generated or analyzed during this study are included in this published article.
